# Reprogramming secretion systems in *Klebsiella quasipneumoniae*: from pathogenic functions to candidate industrial secretion chassis

**DOI:** 10.3389/fbioe.2026.1940225

**Published:** 2026-10-23

**Authors:** Yongli Li, Lixia Liu, Jian Zhang, Guoguang Wei, Long Zhang, Yuanjie Lu, Peiyong Qin, Zhanying Liu

**Affiliations:** 1 Inner Mongolia Key Laboratory of Synthetic Biology, Inner Mongolia University of Technology, Hohhot, Inner Mongolia, China; 2 College of Chemical Engineering, Inner Mongolia University of Technology, Hohhot, Inner Mongolia, China; 3 State Key Laboratory of Green Biomanufacturing, Beijing University of Chemical Technology, Beijing, China

**Keywords:** biocontainment, cell factory, CRISPR genome editing, *Klebsiella* quasipneumoniae, metabolic engineering, secretion systems, signal peptide engineering

## Abstract

Efficient extracellular secretion remains a major bottleneck in microbial cell factories, motivating the search for chassis organisms with native high-throughput transport capacity. *Klebsiella quasipneumoniae* (Kqp), a recently delineated member of the *Klebsiella pneumoniae* species complex, encodes diverse secretion-related systems, including RND-type efflux pumps, type II and type VI secretion systems (T2SS/T6SS), outer membrane vesicle (OMV) pathways, and capsular polysaccharide (CPS) export machinery. In clinical settings, these systems contribute to opportunistic pathogenicity, antimicrobial resistance, and host adaptation; in biotechnology, they may provide transport modules that can be detoxified and redirected for extracellular production. This review examines the feasibility of reprogramming Kqp from a pathogen-related bacterium into a safe industrial secretion chassis. We summarize the molecular architecture, substrate-recognition logic, and regulatory networks of major secretion pathways, then discuss CRISPR-Cas-driven removal of virulence and resistance determinants, biocontainment, and synthetic-biology strategies for reallocating secretory capacity. We further survey potential applications in bulk chemicals, human milk oligosaccharides, recombinant proteins, and industrial enzymes. Finally, we identify key barriers to industrial translation, including growth-secretion energetic trade-offs, periplasmic protein misfolding, endotoxin-associated downstream processing, OMV heterogeneity, scale-up robustness, and quantitative safety validation. Establishing Kqp as an industrial chassis will require evidence-tiered strain selection, quantitative secretion-capacity measurement, regulatory decoupling of resistance-associated pumps, and One Health-aligned safety assessment. Direct evidence in *K. quasipneumoniae* remains limited; therefore, this review distinguishes direct Kqp observations from studies in other KpSC members or *K. oxytoca*, evidence from non-Klebsiella hosts, and forward-looking proposals. OMV yield is considered separately from lipid A/endotoxin-related properties.

## 1 Introduction

Microbial cell factories convert renewable carbon sources into fuels, chemicals, and protein pharmaceuticals. The techno-economic viability of these processes is determined not only by intracellular biosynthetic efficiency but critically by the efficiency with which products are transported across the cell membrane into the extracellular medium. When target products—particularly recombinant proteins and hydrophobic small molecules—accumulate intracellularly, metabolic burden triggers feedback inhibition that thermodynamically caps product titers, while non-specific degradation by intracellular proteases further erodes functional yields. Cell disruption and chromatographic purification account for 50%–80% of total production costs (Karyolaimos and de Gier, 2021). Together, these three constraints constitute the primary economic bottleneck in downstream bioprocessing. Active secretion of products into the extracellular medium represents an effective strategy for simultaneously addressing all three constraints: secretion removes the concentration threshold imposed by feedback inhibition, enabling continuous fermentation with *in situ* product recovery (ISPR); downstream purification is simplified to direct recovery from the supernatant, circumventing the complex workflows of cell lysis and host protein removal; and the extracellular environment provides a chemical microenvironment more conducive to the correct folding of proteins requiring oxidative conditions, such as industrial enzymes with disulfide bonds (Connor et al., 2024). For these reasons, constructing efficient microbial secretion chassis has become a core strategic objective in synthetic biology and industrial biotechnology (Burdette et al., 2018).

Achieving this goal presupposes that the chassis microorganism possesses efficient transmembrane protein transport hardware. A notable mismatch exists, however, between the native secretory capacities of current mainstream industrial chassis and their industrial prominence. Protein secretion in *Escherichia coli* relies principally on the Sec translocon and a limited number of type I secretion systems (T1SS), achieving only moderate extracellular accumulation for most heterologous proteins, and lacks dedicated machinery for actively extruding periplasmic proteins across the outer membrane (Karyolaimos and de Gier, 2021). *Bacillus* subtilis, as a Gram-positive bacterium, enjoys the advantage of direct Sec-pathway secretion into the extracellular milieu in the absence of an outer membrane; however, its substrate spectrum is naturally biased toward specific enzyme classes such as proteases and amylases, and secretion efficiency under high-cell-density fermentation is limited by signal peptidase turnover rates (Ferrando et al., 2024). *Saccharomyces* cerevisiae faces the physical barrier of the cell wall: even when proteins are successfully translocated into the periplasmic space, the diffusion rate of macromolecular proteins across the 100–200 nm cell wall layer is extremely low, constituting a fundamental bottleneck for extracellular release (Xu et al., 2021). These constraints have motivated sustained efforts in the synthetic biology community to identify alternative chassis microorganisms natively equipped with high-throughput secretion machinery. The ideal candidate would simultaneously possess diverse protein secretion systems, efficient transmembrane transport hardware, endogenous tolerance to fermentation products, and well-developed genetic tools. It is against these four criteria that the genus *Klebsiella* has entered the purview of industrial microbiologists.

Before delving into the secretory biology of *Klebsiella*, a framing question merits examination: given the availability of safe, mature, and genetically tractable industrial chassis such as *B. subtilis*, *Corynebacterium glutamicum*, *E. coli* K-12, and *S. cerevisiae*, what rationale supports the selection of a microorganism that shares core genomic content with human pathogens as an industrial chassis? *B. subtilis* is a widely recognized Generally Regarded as Safe (GRAS) strain; its direct Sec-pathway secretion capacity and CRISPR-based engineering of the entire secretion pathway have been validated in hyper-secreting mutants, delivering gram-scale industrial enzyme production (Ferrando et al., 2024). *C. glutamicum*, as the dominant workhorse of the amino acid industry, benefits from decades of safe fermentation history and mature industrial scale-up experience. *E. coli* K-12 is the model chassis with the richest repertoire of regulatory elements and the most comprehensive genetic toolkit in synthetic biology. *S. cerevisiae* offers the unique advantages of a eukaryotic expression system with post-translational modification capacity. Choosing Kqp entails accepting higher regulatory hurdles, public perception risks associated with a pathogen-related organism, and a genetic toolchain that remains under development. These additional costs are justified only when the distinctive advantages of Kqp translate into decisive competitiveness. Specifically, the differentiating value of Kqp is concentrated in three application scenarios: (i) Bulk chemical production requiring high-throughput transmembrane efflux—products such as 1,3-propanediol and 2,3-butanediol impose growth inhibition on chassis cells as a primary industrialization bottleneck; high 1,3-PDO and 2,3-BDO titers reported for *Klebsiella* strains demonstrate fermentation performance but do not identify AcrAB-TolC or OqxAB as the export route; the cited pump studies primarily concern antimicrobial or stress-associated phenotypes (Bhogal et al., 2025; Gravey et al., 2024; Zhang et al., 2023). (ii) Applications requiring OMV-mediated encapsulating secretion—OMVs, as natural nanoliposome carriers, hold considerable promise in vaccine delivery, drug carriers, and nanobioreactors (Besançon et al., 2026; Jiang et al., 2022; Weyant et al., 2023; Szöllősi et al., 2023); OMV biogenesis is an endogenous process in Kqp, obviating the need to introduce heterologous OMV production pathways. (iii) Efficient extracellular release of recombinant proteins requiring coordinated multi-secretion-system operation—*Klebsiella* studies support T2SS- and OMV-associated extracellular transport, whereas T6SS is principally a contact-dependent intercellular effector-delivery apparatus rather than a conventional culture-supernatant secretion route (Li Y. et al., 2023; Cianciotto and White, 2017; Nivaskumar and Francetic, 2014; Storey et al., 2020; Liu et al., 2017). In these three scenarios, the efficiency gains conferred by the secretion hardware of the Kqp chassis may reasonably be expected to outweigh the incremental engineering and regulatory costs. Conversely, when the target product imposes no special demands on secretion hardware, traditional GRAS chassis remain the more economical and lower-risk choice. Recent comparative and One Health genomic surveys further show that KpSC-associated lineages from clinical, livestock, environmental, and dairy reservoirs harbor heterogeneous virulence and resistance repertoires, including emerging *Klebsiella* variicola outbreaks (Pustam et al., 2023; Motlhaping et al., 2026; Legese et al., 2025; González-Espinosa et al., 2025; Prinčič et al., 2024).

The genus *Klebsiella* exhibits a distinctive, multidimensional appeal in this context. At the level of secretion hardware, its genome encodes native secretion apparatuses spanning multiple dimensions. The large-scale biosynthesis and transmembrane export of capsular polysaccharide (CPS) and extracellular polysaccharide (EPS) depend on the *Wzx*/*Wzy*-dependent assembly line coupled with the *Wza*-*Wzc* outer membrane translocation complex, establishing capsule assembly and surface export but not, by itself, industrial secretion throughput (Pan et al., 2015; Guerra et al., 2026; Xu et al., 2024; Whitfield et al., 2020). The T2SS (Pul system) natively secretes pullulanase, amylase, and lipase—enzymes of substantial industrial demand (Li Y. et al., 2023; Cianciotto and White, 2017; Nivaskumar and Francetic, 2014). *Klebsiella* AcrAB-TolC and related RND-pump studies establish antimicrobial-resistance and stress-associated functions; their quantitative contribution to Kqp fermentation-product tolerance has not been demonstrated (Bhogal et al., 2025; Gravey et al., 2024; Zhang et al., 2023; Nikaido, 2009; Wilhelm and Pos, 2024; Ni et al., 2020; Padilla et al., 2010; Nasralddin et al., 2025). OMVs deliver proteins, nucleic acids, and metabolites to the extracellular environment via encapsulating transport, providing a natural molecular template for nanoliposome encapsulation strategies (Hussein et al., 2023; Dell'Annunziata et al., 2021; Varol et al., 2025; Garling et al., 2025). From a safety perspective, the taxonomy of the genus *Klebsiella* has undergone fundamental revision over the past decade. *K. quasipneumoniae* (KpII) has been reclassified as a distinct species separate from traditional *K. pneumoniae*, and together with *K. pneumoniae* sensu stricto (KpI) and *K. variicola* (KpIII) constitutes the KpSC (Brisse et al., 2014; Rodrigues et al., 2018). Multiple genomic epidemiological studies have revealed that KpSC isolates from non-clinical environments (such as bovine mastitis-associated strains) carry very few antimicrobial resistance genes and human virulence factors (Biggel et al., 2025; Bray et al., 2025). Such environmental genotypes combine the metabolic flexibility and secretory capacity of the species complex while lacking the full complement of human virulence determinants in their genomes, making non-clinical origin a useful provenance descriptor rather than evidence of an intrinsically safe industrial phenotype. Accurate species identification—via whole-genome sequencing and validated phylogenomic workflows (Brisse et al., 2014; Galib and Nguyen, 2025), PCR-based marker detection (Galib and Nguyen, 2025), or validated MALDI-TOF workflows (Rodrigues et al., 2018) — thus acquires dual strategic value: clinically, it supports correct epidemiological surveillance; industrially, it guides the rational selection of benign, high-producing chassis starting strains from the natural diversity of the KpSC.

Published *Klebsiella* studies describe secretion and transport systems with distinct natural functions; they do not establish a single pre-configured industrial high-throughput network (Ring et al., 2023). The *Klebsiella* secretion apparatus encompasses protein secretion systems (T1SS–T6SS), RND efflux pumps, OMV biogenesis pathways, and CPS/EPS export machinery, constituting a functionally context-dependent molecular network (Paczosa and Mecsas, 2016; Yang et al., 2023). In the context of host infection, T6SS injects bactericidal effector proteins into competing microbiota to secure ecological niches (Bray et al., 2025; Li W. et al., 2024; Storey et al., 2020; Mohamed et al., 2023; Liu et al., 2023; Zhang et al., 2022; Liu et al., 2017; Haddadi et al., 2025); AcrAB-TolC expels diverse structurally unrelated antibiotics, conferring multidrug-resistant (MDR) phenotypes (Bhogal et al., 2025; Gravey et al., 2024; Zhang et al., 2023; Nikaido, 2009; Wilhelm and Pos, 2024; Ni et al., 2020; Padilla et al., 2010; Nasralddin et al., 2025; Zdarska et al., 2026); OMVs disseminate carbapenemases and virulence factors among bacterial communities (Hussein et al., 2023; Dell'Annunziata et al., 2021; Tang et al., 2023); and the thick CPS layer counteracts phagocytosis and complement attack through physical exclusion and electrostatic repulsion (Guerra et al., 2026; Xu et al., 2024; Whitfield et al., 2020). Their redirection toward industrial products remains a forward-looking concept in this review rather than a demonstrated Kqp property (Burdette et al., 2018). T6SS is a contractile, contact-dependent nanomachine that delivers effectors into neighboring cells; the cited literature does not establish it as a route for bulk product accumulation in culture supernatant (Liu et al., 2017; Cherrak et al., 2021; Flaugnatti et al., 2021). For T2SS, Sec/Tat signal peptides govern inner-membrane targeting, whereas outer-membrane export additionally depends on periplasmic folding and recognition of mature-substrate features by the T2SS machinery (Karyolaimos and de Gier, 2021; Li Y. et al., 2023; Cianciotto and White, 2017; Nivaskumar and Francetic, 2014; Eom et al., 2024; Ghasemi et al., 2022; Zhang and Duan, 2023). The above analysis crystallizes into the core engineering proposition of this review: preserve the transport infrastructure, eliminate harmful cargoes, and redirect secretory flux toward high-value products. The knowledge base required to realize this proposition is distributed across four progressive levels: (i) systematic molecular-level analysis of the structural architecture, substrate recognition logic, and transcriptional regulatory networks of the Kqp secretion machinery (Chapter 2); (ii) utilization of CRISPR-Cas genome editing tools—from the dual-plasmid pCasKP-pSGKP system (Wang et al., 2018) to the single-plasmid modular RECKLEEN platform (Elsayed et al., 2025) and attenuated gRNA strategies (Collias et al., 2023; Yao et al., 2022) — to precisely delete virulence effector proteins and horizontally transferable resistance determinants while preserving the structural integrity of secretion channel components (Chapter 3); (iii) application of synthetic biology enabling technologies including signal peptide engineering, efflux pump substrate reprogramming, and OMV chassis engineering to actively enhance secretory flux and substrate specificity (Chapter 4); (iv) quantitative assessment of the practical application potential and current technical bottlenecks of engineered Kqp chassis in bulk chemicals (1,3-propanediol/2,3-butanediol), high-value carbohydrates (2′-fucosyllactose and rare sugars), and recombinant protein and industrial enzyme secretion (Chapter 5). Finally, this review systematically discusses four fundamental bottlenecks facing the field—the energetic cost of secretion and the growth–secretion trade-off, the periplasmic heterologous protein misfolding–degradation vicious cycle, batch-to-batch heterogeneity of OMV products, and quantitative safety validation of biocontainment systems—and outlines a five-to-ten-year research agenda spanning dynamic metabolic control, AI-guided rational design of transporters, and One Health-standardized safety assessment frameworks (Chapter 6). Throughout this review, direct Kqp evidence, evidence from other *Klebsiella* taxa, non-*Klebsiella* analogies, and prospective concepts are identified separately.

## 2 Secretion mechanisms and antimicrobial resistance/virulence networks of Kqp

### 2.1 Efflux pumps and multidrug resistance

Efflux pumps constitute the first biochemical line of defense in Gram-negative bacterial antibiotic resistance (Nikaido, 2009; Wilhelm and Pos, 2024). Figure 1 summarizes secretion and transport classes discussed in *Klebsiella* and broader Gram-negative literature; not every depicted system has been directly characterized in Kqp (Ni et al., 2020; Zdarska et al., 2026). Figure 1 depicts the spatial organization and functional division of labor among eight categories of secretion/efflux systems within the cell envelope. In the dual-membrane architecture of Gram-negative bacteria, each category of secretion apparatus has evolved a distinctive transmembrane strategy and energy-coupling mechanism: T1SS (Figure 1A) is assembled from an inner membrane ABC transporter, a periplasmic membrane fusion protein (MFP), and an outer membrane TolC-like channel, driving one-step substrate translocation across both membranes via ATP hydrolysis without a periplasmic intermediate (Yang et al., 2023). T2SS (Figure 1B) employs Sec/Tat-dependent two-step secretion: after precursor proteins fold in the periplasm, they are extruded through the PulD secretin channel by a pseudopilus piston mechanism driven by the PulE ATPase (Li Y. et al., 2023; Cianciotto and White, 2017; Nivaskumar and Francetic, 2014). T3SS (Figure 1C) is an injectisome associated with effector delivery in strains that encode the system (Yang et al., 2023). T4SS (Figure 1D), via its VirB/D4 system, mediates conjugative transfer and is a key molecular conduit for the horizontal dissemination of resistance plasmids and virulence gene islands (Yang et al., 2023). Classical type Va secretion (Figure 1E) uses Sec-dependent inner-membrane translocation and a C-terminal β-barrel with Bam-complex assistance; this general mechanism does not establish Kqp-specific prevalence (Bernstein, 2019). T6SS (Figure 1F) is a contractile injection nanomachine homologous to bacteriophage tails, in which contraction of the TssB/C outer sheath drives the Hcp inner tube–VgrG/PAAR tip complex to puncture target cells (Storey et al., 2020; Liu et al., 2017). The RND efflux pump AcrAB-TolC (Figure 1G) is the core molecular machine underlying the MDR phenotype of *Klebsiella*; through the functional rotation mechanism of the AcrB trimer (Loose→Tight→Open three-state cycle), it utilizes the proton motive force to expel drugs, with hydrophobic pocket residues (F136, F178, F614, F627, and V671) in its distal binding pocket forming a hydrophobic trap that recognizes structurally diverse substrates (Zhang et al., 2023). OMV budding and CPS/EPS export pathways (Figure 1H) represent two distinctive modes of encapsulating secretion: OMVs are 20–250 nm spherical lipid bilayer nanovesicles formed by outer membrane bulging, while CPS is transported to the cell surface via the Wzx/Wzy/Wza polysaccharide assembly–export pipeline (Pan et al., 2015; Guerra et al., 2026; Dell'Annunziata et al., 2021). The following sections discuss in depth the molecular mechanisms and engineering potential of these secretion apparatuses in resistance, pathogenesis, and industrial applications.

**FIGURE 1:**
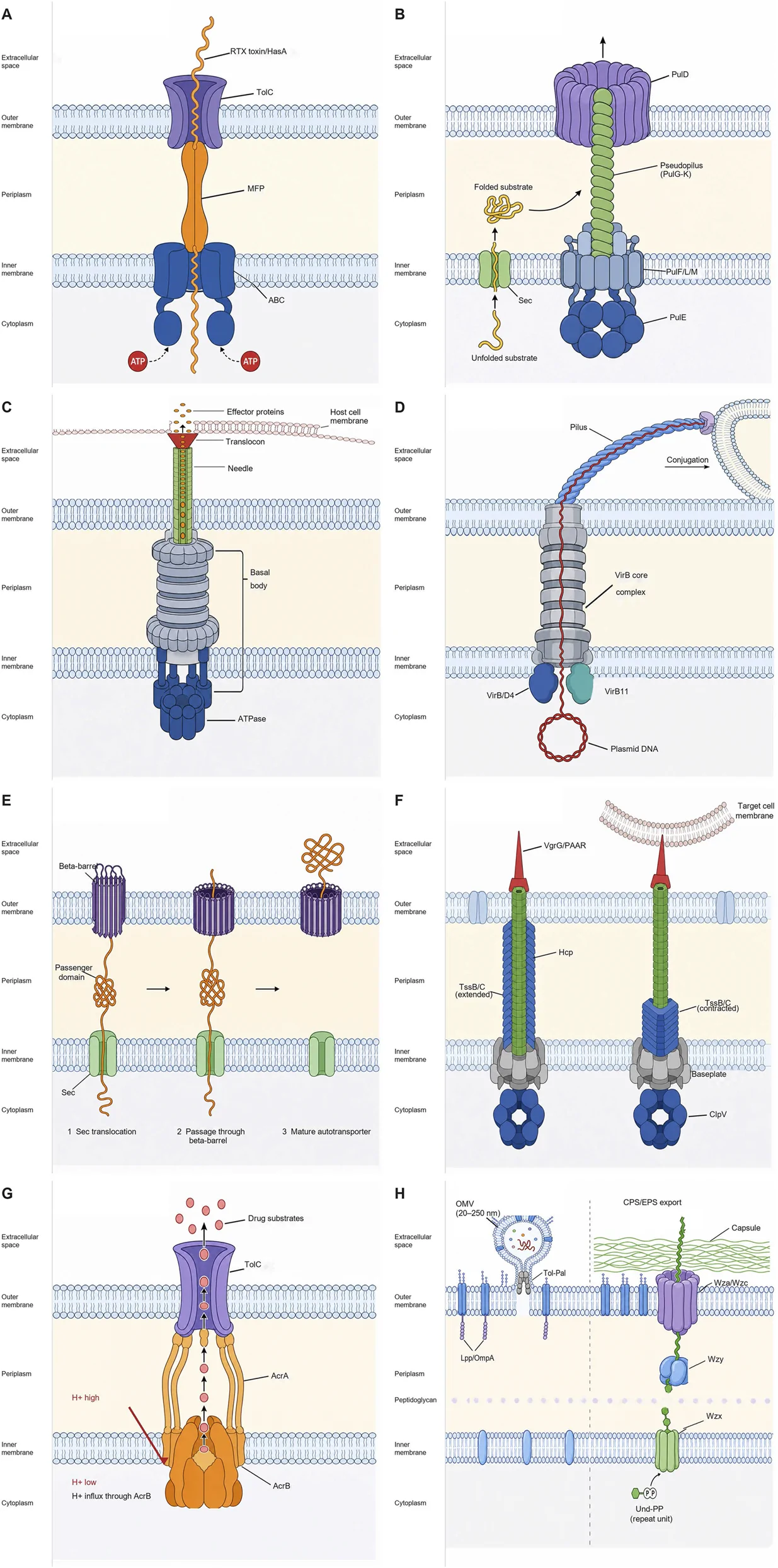
Mechanistic overview of secretion and transport systems discussed for *Klebsiella* and other Gram-negative bacteria. **(A)** T1SS: ABC transporter–MFP–OMP one-step export. **(B)** T2SS (Pul): Sec/Tat-dependent inner-membrane translocation followed by PulD/PulG ∼ K/PulF-L-M/PulE-mediated export of folded periplasmic substrates. **(C)** T3SS: injectisome. **(D)** T4SS: VirB/D4 conjugative-transfer system. **(E)** Classical type Va secretion: N-terminal passenger and C-terminal β-barrel. **(F)** T6SS: contact-dependent TssB/C–Hcp–VgrG/PAAR apparatus. **(G)** AcrAB-TolC RND efflux pump. **(H)** OMV budding and Wzx/Wzy/Wza/Wzc-dependent capsule export. The diagram is schematic and does not imply that every pathway has been directly characterized in Kqp. Data sources: (Burdette et al., 2018; Zhang et al., 2023; Li Y. et al., 2023; Cianciotto and White, 2017; Nivaskumar and Francetic, 2014; Pan et al., 2015; Guerra et al., 2026; Xu et al., 2024; Whitfield et al., 2020; Hussein et al., 2023; Dell'Annunziata et al., 2021; Yang et al., 2023; Storey et al., 2020; Liu et al., 2017; Zhu et al., 2025; Bernstein, 2019).

The AcrAB-TolC tripartite complex is the functionally and clinically most significant system within the RND family (Wilhelm and Pos, 2024; Padilla et al., 2010). Zhang et al. (Zhang et al., 2023) determined near-atomic-resolution structures of KpAcrB in the apo state and erythromycin-bound state using single-particle cryo-electron microscopy (cryo-EM) (Figure 2), revealing two key features: KpAcrB shares 91% amino acid sequence identity with its *E. coli* homolog and adopts the conserved HAE-RND fold. Figure 2 presents, in four panels, the assembly architecture, transport mechanism, drug recognition, and transcriptional regulation of this pump. At the level of overall architecture (Figure 2A), the inner membrane transporter AcrB is embedded as a homotrimer in the inner membrane, with each protomer comprising 12 transmembrane α-helices and a periplasmic domain extending approximately 70 Å into the periplasm, with a total molecular mass of ∼113.5 kDa. The periplasmic membrane fusion protein AcrA transmits conformational changes from AcrB to TolC via an α-helical hairpin domain. TolC forms a trimeric α-helical tunnel approximately 140 Å in length, with a funnel-shaped extracellular opening of ∼30–40 Å internal diameter (Zhang et al., 2023; Wilhelm and Pos, 2024; Zdarska et al., 2026). In terms of transport mechanism (Figure 2B), each AcrB protomer cycles counterclockwise among three conformations—substrate access (Loose), binding (Tight), and extrusion (Open) — driven by the proton gradient (ΔμH^+^) across the inner membrane. With respect to drug recognition (Figure 2C), the structure-derived view based on PDB 8FFS shows that five hydrophobic pocket residues (F136, F178, F614, F627, and V671) in the distal drug-binding pocket form a hydrophobic binding environment through hydrophobic interactions, accommodating fluoroquinolones, tetracyclines, macrolides, β-lactams, bile salts, and diverse organic solvents via a multi-specificity binding mode, explaining the exceptionally broad substrate spectrum of AcrB (Zhang et al., 2023).

**FIGURE 2:**
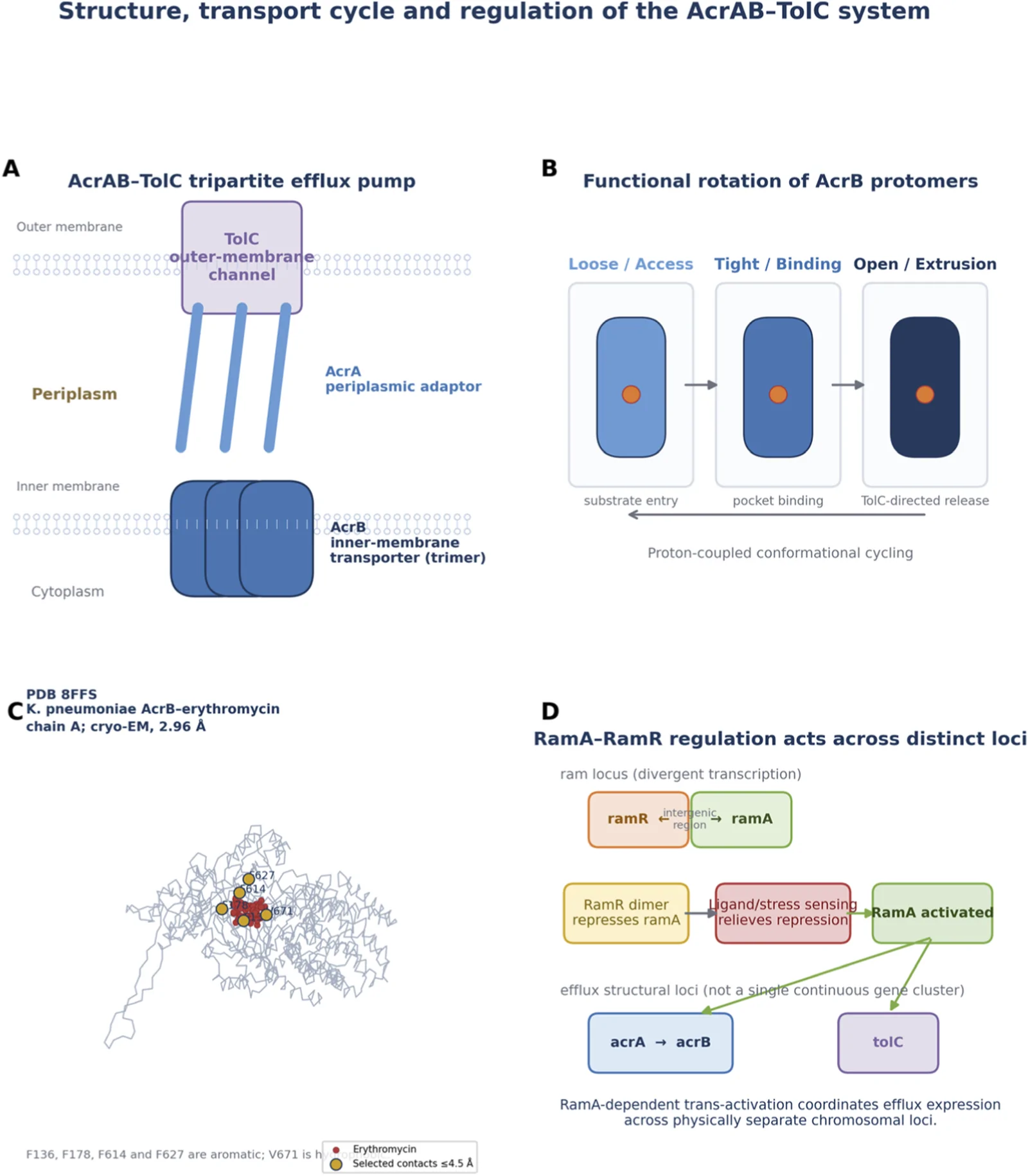
Structure and drug recognition mechanism of the AcrAB-TolC tripartite efflux pump. **(A)** Side view of the tripartite architecture: AcrB-AcrA-TolC. **(B)** Functional rotation three-state cycle of AcrB (Loose→Tight→Open). **(C)** Structure-derived view of erythromycin-bound *K. pneumoniae* AcrB (PDB 8FFS; 2.96 Å), showing the ligand and residues F136/F178/F614/F627/V671. The four phenylalanines are aromatic; V671 is hydrophobic. **(D)** RamA-RamR transcriptional regulatory axis: RamR repression → antibiotic binding-induced dissociation → RamA activation of the separately located *acrAB* operon and *tolC* gene. Data sources: (Bhogal et al., 2025); (Gravey et al., 2024); (Zhang et al., 2023); (Nikaido, 2009; Wilhelm and Pos, 2024; Ni et al., 2020; Padilla et al., 2010; Nasralddin et al., 2025).

Expression of AcrAB-TolC is under the strict control of the RamA-RamR regulatory axis (Figure 2D) (Gravey et al., 2024; Nasralddin et al., 2025). RamR, a TetR-family transcriptional repressor, binds as a homodimer to the intergenic region between ramR and ramA, physically blocking RNA polymerase from initiating ramA transcription. When antibiotic or bile acid molecules enter the cell and bind to the ligand-binding domain of RamR, they induce a conformational change that causes RamR to dissociate from DNA; derepression of *ramA* then drives overexpression of the *acrAB* operon and the separately located *tolC* gene. Gravey et al. (Gravey et al., 2024) conducted a combined genomic and transcriptomic analysis of 443 extended-spectrum β-lactamase (ESBL)-producing Enterobacterales isolates from French ICUs and demonstrated that 13.1% of *K. pneumoniae* and 11.8% of *Enterobacter cloacae* complex strains exhibited a joint resistance phenotype (t2c2) to temocillin, tigecycline, ciprofloxacin, and chloramphenicol, a phenotype that was completely reversed by the efflux pump inhibitor Phe-Arg-β-naphthylamide (PAβN) in all tested strains. Among 31 t2c2-positive strains, 30 carried functional mutations in RamR or the ramAR intergenic region, and transcriptomic analysis confirmed overexpression of ramA, acrA, and tolC (Gravey et al., 2024). These data indicate an evolutionary trajectory in which RamR inactivation represents the shortest path for *Klebsiella* to acquire efflux-mediated multidrug resistance under antibiotic selection pressure. Bhogal et al. (Bhogal et al., 2025) further demonstrated that AcrAB-TolC also participates in the efflux of host-derived antimicrobial compounds, including bile acids and short-chain fatty acids, directly promoting intestinal colonization, indicating that the physiological functions of this pump extend beyond mere antibiotic extrusion.

In addition to AcrAB-TolC, OqxAB is another RND-type efflux pump that makes an independent contribution to fluoroquinolone resistance. Amereh et al. (Amereh et al., 2023a) reported that among clinical *K. pneumoniae* isolates from Iran, the carriage rates of oqxA and oqxB reached 95% and 98%, respectively, with a ciprofloxacin resistance rate of 89%, and a significant association between oqxA expression levels and ciprofloxacin resistance (Table 1; Figure 3). Figure 3 presents a conceptual Sankey diagram tracing the flow paths from the three KpSC species through different resistance mechanisms to various antibiotic resistance outcomes: the left column represents the three KpSC species (KpI red/KpII orange/KpIII green), with node heights serving as qualitative schematic elements; the middle column shows five categories of resistance mechanisms—efflux pump overexpression, porin loss (OmpK35/36), carbapenemases (KPC/NDM/OXA-48), ESBLs (CTX-M/SHV), and CPS/EPS barrier; the right column represents six antibiotic resistance outcomes. At the flow-ribbon level, this figure illustrates two qualitative relationships: the thickest flow ribbons from KpI through efflux pump overexpression and carbapenemase pathways to fluoroquinolone and β-lactam resistance are the most prominent, illustrating clinically relevant resistance pathways without quantifying their prevalence (Voellmy et al., 2022); simultaneously, the efflux pump overexpression node radiates to nearly all six resistance endpoints, indicating the cross-class breadth of efflux-mediated resistance. A multiplicative effect exists between active efflux and the passive barrier imposed by porin downregulation: the major porins OmpK35 and OmpK36 regulate the passive influx of β-lactams and fluoroquinolones (Zdarska et al., 2026); OmpK36 loss is directly associated with elevated carbapenem MICs, and GD/TD dipeptide insertions in the loop three domain can push carbapenem MICs to 256 mg/L (Zdarska et al., 2026; Teng et al., 2024). The superposition of efflux overexpression and porin loss constitutes the core molecular basis of MDR phenotypes in *Klebsiella.*

**TABLE 1:** Overview of major secretion/efflux systems discussed for *Klebsiella* and related Gram-negative systems (including components, substrates, energy sources, distribution, pathogenic roles, and engineering potential). References: (Amereh et al., 2023a); (Besançon et al., 2026); (Bhogal et al., 2025); (Bray et al., 2025); (Cherrak et al., 2021); (Connor et al., 2024); (Dai Y. et al., 2025); (Li Y. et al., 2023); (Flaugnatti et al., 2021); (Galib and Nguyen, 2025); (Gravey et al., 2024); (Hussein et al., 2023); (Ji et al., 2026); (König et al., 2021); (Li Q. et al., 2023); (Li W. et al., 2024); (Dell'Annunziata et al., 2021); (Bray et al., 2025); (Oh et al., 2018); (Pan et al., 2015); (Yang et al., 2023); (Guerra et al., 2026); (Wang et al., 2018); (Zdarska et al., 2026); (Zhang et al., 2023); (Zhu et al., 2025); (Cianciotto and White, 2017; Nivaskumar and Francetic, 2014; Xu et al., 2024; Whitfield et al., 2020; Bray et al., 2025; Storey et al., 2020; Mohamed et al., 2023; Liu et al., 2023; Zhang et al., 2022; Liu et al., 2017; Haddadi et al., 2025; Tang et al., 2023; D'Souza et al., 2021). Direct Kqp evidence is identified separately from evidence in other hosts.

System	Type	Key components	Natural substrates/function	Energy	Evidence distribution	Pathogenic role	Evidence-based application boundary	References
AcrAB-TolC	RND efflux pump	AcrB (IM) + AcrA + TolC (OM)	Fluoroquinolones, tetracyclines, β-lactams, bile salts, solvents	PMF (H^+^)	*K. pneumoniae* and other Enterobacterales	Core of MDR; intestinal colonization	Resistance/stress-associated efflux; 1,3-PDO and 2,3-BDO export not demonstrated	(Bhogal et al., 2025; Gravey et al., 2024; Zhang et al., 2023; Ni et al., 2020; Padilla et al., 2010; Nasralddin et al., 2025)
OqxAB	RND efflux pump	OqxB + OqxA + TolC-like	Fluoroquinolones (ciprofloxacin), chloramphenicol	PMF (H^+^)	Clinical *K. pneumoniae* isolates (source-specific)	Quinolone resistance	Antimicrobial efflux; diol-export role not demonstrated	(Amereh et al., 2023a); (Zdarska et al., 2026)
T1SS	One-step secretion	ABC transporter + MFP + OMP	RTX toxins, HasA hemoprotein	ATP	Variable across strains	Cytotoxin export	One-step heterologous protein secretion	(Yang et al., 2023)
T2SS (Pul)	Two-step secretion	PulD + pseudopilus + PulF/L/M + PulE	Pullulanase, lipase, protease	ATP (PulE) + PMF	*K. oxytoca/K. pneumoniae* Pul models	Extracellular enzymes; tissue invasion	PulA benchmark; signal peptide alone does not specify T2SS cargo	(Li et al., 2023a; Cianciotto and White, 2017; Nivaskumar and Francetic, 2014; Zhang and Duan, 2023; Guilvout et al., 2024; Pugsley et al., 1986)
T3SS	Injectisome	Needle complex (IM + OM) + translocon pore	Effector proteins → host cytoplasm	ATP	Strain-dependent in Gram-negative pathogens	Host cell manipulation	Not an established Kqp product-secretion platform	(Yang et al., 2023)
T4SS	Conjugative transfer	VirB/D4 complex + pilus	Plasmid DNA, protein-DNA complexes	ATP	Variable across strains	Resistance/virulence plasmid dissemination	Low (needs elimination)	(Yang et al., 2023)
T5SS	Classical type Va secretion	C-terminal β-barrel (OM) + N-terminal passenger domain	Adhesins, proteases, IgA proteases	Sec/Bam-dependent	Widely distributed in Gram-negative bacteria; Kqp prevalence not established	Adhesion, immune evasion	Autotransporter display/secretion precedents exist outside Kqp	(Bernstein, 2019)
T6SS	Contact-dependent injection	TssB/C (sheath) + Hcp + VgrG/PAAR + ClpV	Antibacterial effectors	ATP (ClpV) + mechanical force	Demonstrated in *K. pneumoniae* strains	Interbacterial competition; gut colonization	Competition mechanism; not a bulk culture-supernatant route	(Bray et al., 2025; Li et al., 2024a; Storey et al., 2020; Mohamed et al., 2023; Liu et al., 2023; Zhang et al., 2022; Liu et al., 2017; Haddadi et al., 2025; Zhu et al., 2025)
OMV	Outer membrane budding	Lpp-OmpA-PG anchoring; Tol-Pal	β-lactamases, plasmid DNA, LPS	Turgor pressure (passive)	*K. pneumoniae* and other Gram-negative bacteria	Enzyme delivery; HGT; immune evasion	Delivery applications in source hosts; endotoxin is a separate attribute	(Besançon et al., 2026; Jiang et al., 2022; Weyant et al., 2023; Szöllősi et al., 2023; Hussein et al., 2023; Dell'Annunziata et al., 2021; Tang et al., 2023; Li et al., 2023b; Leitner et al., 2013; Kim et al., 2009; Ruiz-Moreno et al., 2025)
CPS/EPS	Polysaccharide assembly-export	WbaP/WcaJ + GTs + Wzx + Wzy + Wza/Wzc	CPS (>79 serotypes); EPS	Und-P turnover + ATP (Wzc)	KpSC; locus- and serotype-dependent	Anti-phagocytosis; biofilm; desiccation tolerance	Capsule export does not demonstrate HMO-flux redirection	(Pan et al., 2015; Guerra et al., 2026; Xu et al., 2024; Whitfield et al., 2020; Dai et al., 2025b)

**FIGURE 3:**
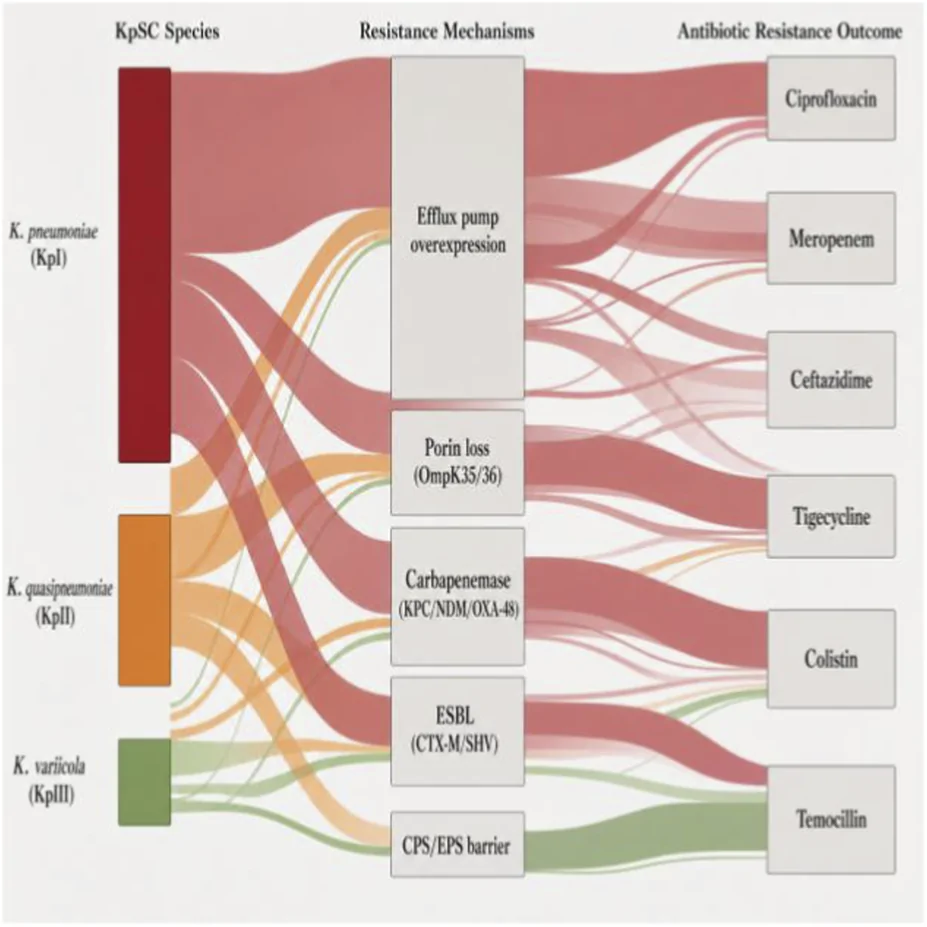
Conceptual Sankey diagram of KpSC species–resistance mechanism–antibiotic resistance relationships. Left column: *K. pneumoniae* sensu stricto (KpI), K. quasipneumoniae (KpII), and K. variicola (KpIII). Middle column: efflux-pump overexpression, OmpK35/36 loss, carbapenemases, ESBLs, and the CPS/EPS barrier. Right column: antibiotic-resistance outcomes. Node heights and ribbon widths are qualitative graphical elements and are not scaled to prevalence, clinical-isolation frequency, or measured flux. The diagram is schematic and not to scale. Data sources: (Zdarska et al., 2026; Amereh et al., 2023a; Voellmy et al., 2022).

In terms of β-lactam resistance, the chromosomally encoded *blaOKP* (a class A β-lactamase and the species-defining chromosomal resistance gene of *K. quasipneumoniae*) provides baseline resistance to aminopenicillins and early-generation cephalosporins (Zdarska et al., 2026; D'Souza et al., 2021). Its chromosomal location distinguishes it from plasmid-encoded ESBLs (CTX-M types, SHV-12, etc.) and carbapenemases (KPC-2, NDM-1, OXA-48-like, etc.) acquired through horizontal gene transfer (Zdarska et al., 2026; Dai L. et al., 2025; F et al., 2026). In KPC-2 and NDM-5 co-producing CRKP strains, Dai et al. (Dai L. et al., 2025) uncovered a two-component adaptive response strategy: amplification of the *blaNDM*-5 plasmid copy number ensured adequate enzyme concentrations, while concurrent 2.1-fold upregulation of *wecC* enhanced biofilm formation by 42%, indicating synergistic coordination among three defense mechanisms—enzymatic inactivation, efflux pump extrusion, and biofilm physical barrier (Dai L. et al., 2025). Beyond patient-to-patient spread, resistance determinants also circulate through healthcare textiles and One Health reservoirs; the co-occurrence of *qnr* genes and OqxAB further illustrates plasmid-linked fluoroquinolone resistance (Cayrou et al., 2025; Guitart-Matas et al., 2026; Amereh et al., 2023b).

### 2.2 Outer membrane vesicle-mediated community resistance

Outer membrane vesicles (OMVs) are spherical lipid bilayer nanostructures, typically 20–250 nm in diameter, that are continuously released from the outer membrane of growing Gram-negative bacteria through a budding process. Their biogenesis involves local dissociation of cross-links between the outer membrane and the peptidoglycan layer—principally maintained by covalent anchoring of Braun’s lipoprotein (Lpp) and non-covalent interactions of OmpA—coupled with turgor pressure-driven outward bulging and membrane scission (Dell'Annunziata et al., 2021; Varol et al., 2025; Garling et al., 2025). In *Klebsiella*, OMV functions extend far beyond passive vesicle shedding; rather, they constitute a sophisticated remote molecular delivery system that extends the effective range of resistance and virulence factors from the individual producing cell to the entire bacterial community.

The most clinically significant function of OMVs is the encapsulation and intercellular dissemination of β-lactamases. Hussein et al. (Hussein et al., 2023) conducted a comparative OMV proteomic analysis of paired *K. pneumoniae* strains susceptible (MIC = 0.5 mg/L) and extremely resistant (MIC ≥128 mg/L) to polymyxin B, comparing treated and untreated OMVs within each strain (Figure 4). The categorical evidence map in Figure 4 summarizes strain-specific responses to polymyxin B. Proteins involved in adhesion, virulence, and envelope-stress responses decreased in susceptible-strain OMVs, whereas proteins associated with LPS biosynthesis, RNA degradation, and nucleotide excision repair increased in resistant-strain OMVs. ArnT abundance decreased after treatment in both strains, with a larger decrease in the susceptible strain. Class A β-lactamases also decreased in OMVs of both strains. These findings support treatment-dependent remodeling of OMV cargo and a proposed decoy role during polymyxin exposure (Hussein et al., 2023). Dell'Annunziata et al. (Dell'Annunziata et al., 2021) further demonstrated that K. pneumoniae-derived OMVs can directly package and transfer plasmid DNA to recipient strains, extending the functional role of OMVs from passive cargo packaging to active mediators of non-conjugative horizontal gene transfer (Dell'Annunziata et al., 2021; Tang et al., 2023). The engineering potential of OMVs is equally noteworthy: Besançon et al. (Besançon et al., 2026) utilized a STING-adjuvanted OMV-gold nanoparticle platform (CNP-KpSTING) to achieve cross-protection against different Kp capsule types in a lethal pneumonia model; the modular design of the same platform has been successfully applied to *P. aeruginosa* (Jiang et al., 2022; Weyant et al., 2023; Szöllősi et al., 2023; Bjånes et al., 2025; Li Q. et al., 2023). Structural analysis of OMV-embedded NadA further indicates that vesicle presentation can influence antigen conformation and immunogenicity (Calvaresi et al., 2026).

**FIGURE 4:**
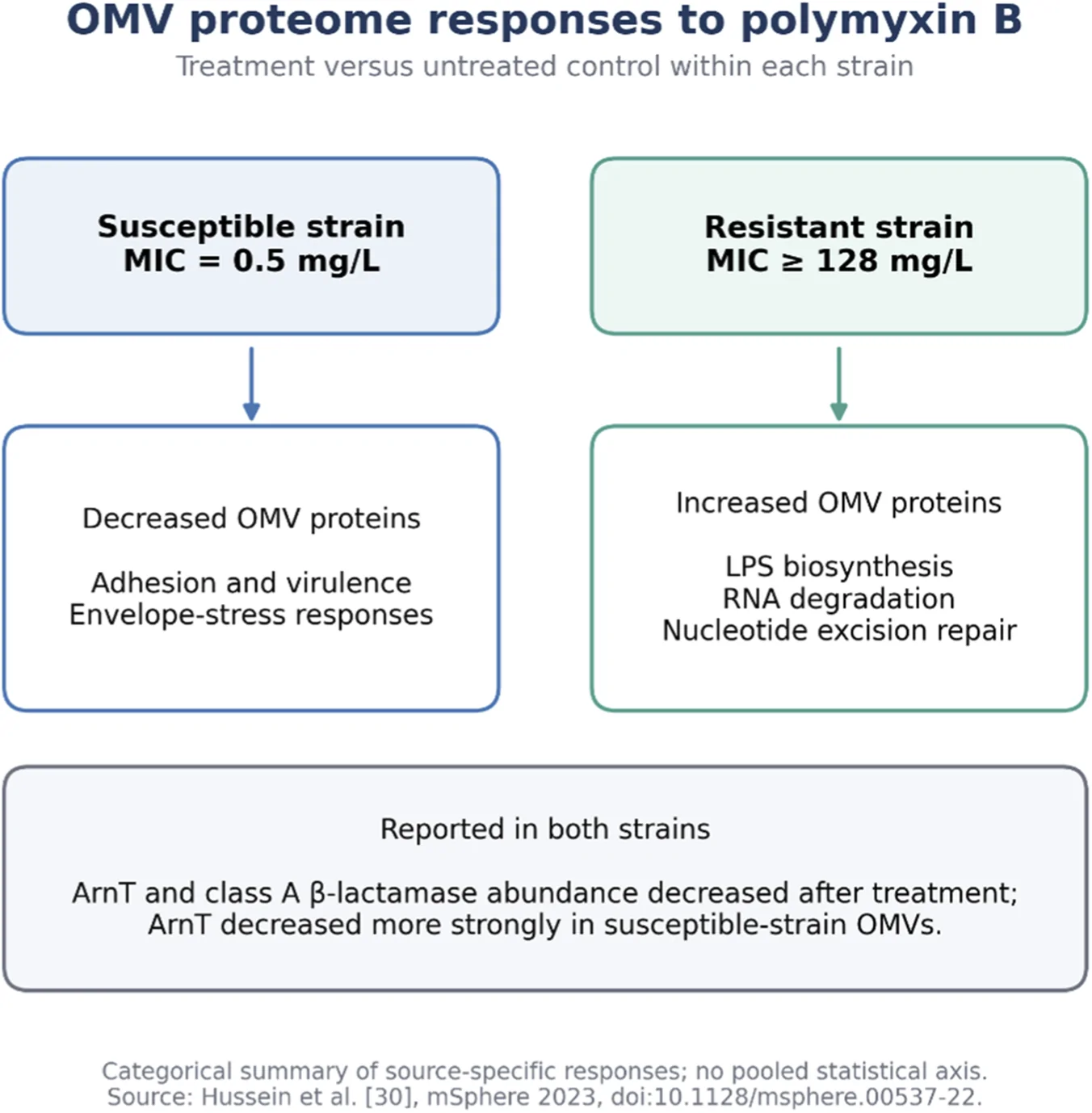
Categorical summary of OMV proteome responses to polymyxin B in susceptible and resistant *K. pneumoniae*. Each response refers to treatment versus the untreated control within the same strain. Functional categories and selected protein changes are summarized without pooled fold-change axes or a common significance threshold. Data source: (Hussein et al., 2023).

### 2.3 Classical protein secretion systems and pathogenicity

T2SS transports folded periplasmic proteins across the outer membrane in *Klebsiella* models (Cianciotto and White, 2017; Nivaskumar and Francetic, 2014). The Pul system of *K. oxytoca* comprises 12–15 proteins organized into an outer-membrane secretin, a periplasmic pseudopilus, an inner-membrane assembly platform, and a cytoplasmic ATPase (Li Y. et al., 2023). Li et al. characterized the interaction between the PulL and PulM periplasmic helices using bacterial two-hybrid and functional assays, supporting a coiled-coil interface critical for secretion (Li Y. et al., 2023). Signal peptides deliver substrates to Sec or Tat across the inner membrane but are not sufficient to specify T2SS export; the folded periplasmic substrate must also be recognized by the T2SS machinery (Cianciotto and White, 2017; Nivaskumar and Francetic, 2014). These studies define the architecture, interaction interfaces, and substrate-recognition constraints of the *Klebsiella* Pul T2SS.

T6SS is a contractile injection nanomachine whose core architecture comprises a contractile outer sheath (copolymer of TssB and TssC proteins), an inner tube (tubular structure formed by stacked Hcp hexameric rings), and a tip complex consisting of the VgrG trimer–PAAR protein spike (Storey et al., 2020; Liu et al., 2017). Li et al. (Li W. et al., 2024) performed whole-genome sequencing of 65 clinical *K. pneumoniae* isolates and confirmed that all strains carry at least one T6SS gene cluster; single-gene knockouts of *hcp* and *vgrG* in the ST11 strain HS11286, combined with transcriptomic analysis, revealed 1,298 commonly upregulated and 1,752 commonly downregulated genes in the two mutants (Figure 5). Figure 5 separates these 65-isolate genomic data from the HS11286 mutant transcriptomic results. Zhu et al. (Zhu et al., 2025) independently profiled T6SS loci and their associated effector-immunity repertoires across *Klebsiella* genomes, revealing variation in their distribution; these data are shown separately from the 65-isolate *cohort*. These studies describe lineage-dependent effector repertoires and experimentally established roles in interbacterial competition (Bray et al., 2025; Mohamed et al., 2023; Liu et al., 2023; Zhang et al., 2022; Liu et al., 2017; Haddadi et al., 2025; Flaugnatti et al., 2021; Zhu et al., 2025). Cherrak et al. (Cherrak et al., 2021) rationally designed a biomimetic cyclic peptide that effectively blocks T6SS assembly and function by targeting the TssK-TssG interface within the T6SS baseplate wedge complex, based on a high-resolution crystal structure. Furthermore, Flaugnatti et al. (Flaugnatti et al., 2021) confirmed that human gut commensal *Klebsiella* can also kill pathogenic bacteria in a T6SS-dependent manner. Collectively, the cited evidence supports T6SS as a contact-dependent competition system, not a conventional route for bulk extracellular product recovery.

**FIGURE 5:**
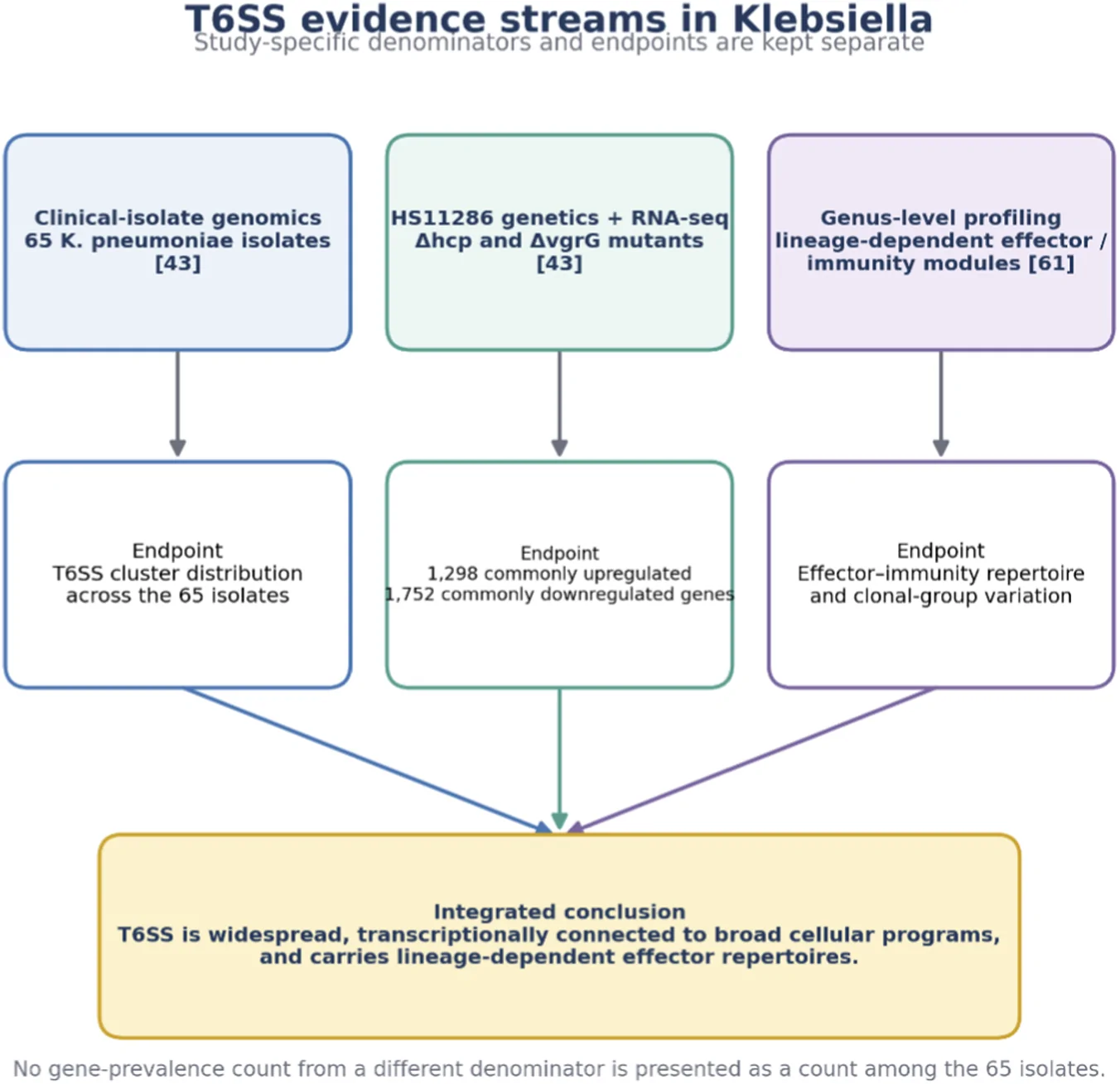
Study-specific evidence for *Klebsiella* T6SS. The left panel summarizes genomic analysis of 65 clinical *K. pneumoniae* isolates (Li W. et al., 2024). The middle panel shows 1,298 commonly upregulated and 1,752 commonly downregulated genes in HS11286 hcp and *vgrG* mutant transcriptomic comparisons (Li W. et al., 2024). The right panel summarizes independent genus-level profiling of T6SS-associated effector-immunity repertoires (Zhu et al., 2025). Transcript counts and genomic prevalence are separate endpoints.

T1SS is the most structurally streamlined protein secretion system, composed of three components—an inner membrane ABC transporter, a membrane fusion protein, and an outer membrane protein—capable of one-step secretion of unfolded substrate proteins directly from the cytoplasm across both membranes into the extracellular environment (Yang et al., 2023). T4SS in *Klebsiella* principally mediates conjugative transfer and intercellular transport of protein-DNA complexes, serving as a key molecular conduit for the horizontal dissemination of resistance plasmids and virulence gene islands (Yang et al., 2023). Classical type Va secretion is well established across Gram-negative bacteria, but the cited general mechanism does not provide a strain-resolved prevalence estimate for Kqp (Bernstein, 2019).

### 2.4 Capsular polysaccharide and extracellular polysaccharide secretion pathways

The polysaccharide capsule is the most conspicuous ultrastructural feature of the genus *Klebsiella*, with at least 79 capsular serotypes (K antigens) synthesized via the *Wzx*/*Wzy*-dependent pathway in the KpSC (Pan et al., 2015; Xu et al., 2024). The *Wzx*/*Wzy*-dependent biosynthesis of CPS is a five-step, membrane-sequenced reaction process: WbaP or WcaJ catalyzes the initiating glycosylation; serotype-specific glycosyltransferases add successive monosaccharide residues in a strictly defined order; the Wzx flippase translocates Und-PP-linked repeat units to the periplasmic side; the Wzy polymerase catalyzes chain elongation on the periplasmic side; and finally, the Wza-Wzb-Wzc ternary complex transports the polysaccharide chains to the cell surface (Guerra et al., 2026; Xu et al., 2024; Whitfield et al., 2020).

The contribution of the capsule to pathogenicity is stage-dependent (Figure 6). Figure 6 uses a five-axis radar chart to compare the phenotypic profile shifts of WT (blue), Δ*Wza* (orange), and *Wzc* mutant (green) strains across five dimensions: biofilm formation, 24 h intracellular proliferation, serum resistance, free EPS production, and capsule thickness. The blue polygon of the WT strain protrudes prominently on the serum resistance and intracellular proliferation axes, yet its biofilm formation capacity is limited. The orange polygon of the Δ*Wza* mutant shifts markedly toward the biofilm formation axis (+++) while the serum resistance axis contracts severely (−), revealing the virulence trade-off effect of the capsule: while capsular polysaccharide, as a physical barrier, prevents complement deposition and phagocyte recognition, it simultaneously hinders bacterial contact with biotic surfaces and intercellular adhesion. Guerra et al. (Guerra et al., 2026) used quantitative data from wza knockout mutants to show that during the intracellular proliferation phase, the encapsulated wild-type strain achieved approximately 33.6-fold CFU proliferation within 24 h, whereas the non-encapsulated mutant reached only approximately 4-fold—a difference of nearly one order of magnitude, clearly demonstrating the protective umbrella role of the capsule during late-stage infection. The green polygon of the Wzc mutant strain dominates on the free EPS production and capsule thickness axes, reflecting the fine-tuning of CPS polymer length and cell surface anchoring efficiency by the phosphorylation state of Wzc protein; the mutant produces excessive low-molecular-weight free EPS but has impaired capacity to assemble a dense capsular layer. Dai et al. (Dai Y. et al., 2025) further revealed in ST11-KL47 CRKP strains that *Wzc* mutation leads to pleiotropic effects including transcriptional downregulation of LPS biosynthesis. Ji et al. (Ji et al., 2026) provided independent evidence from a phage perspective: resistant mutants selected by a two-phage cocktail all carried wzc or wcaJ mutations, resulting in CPS loss and a spectrum of fitness costs. These capsule studies establish pleiotropic effects on envelope and infection-associated phenotypes; they do not demonstrate that *wbaP* or *wcaJ* perturbation redirects nucleotide-sugar precursors to heterologous polysaccharide production (Guerra et al., 2026; Xu et al., 2024).

**FIGURE 6:**
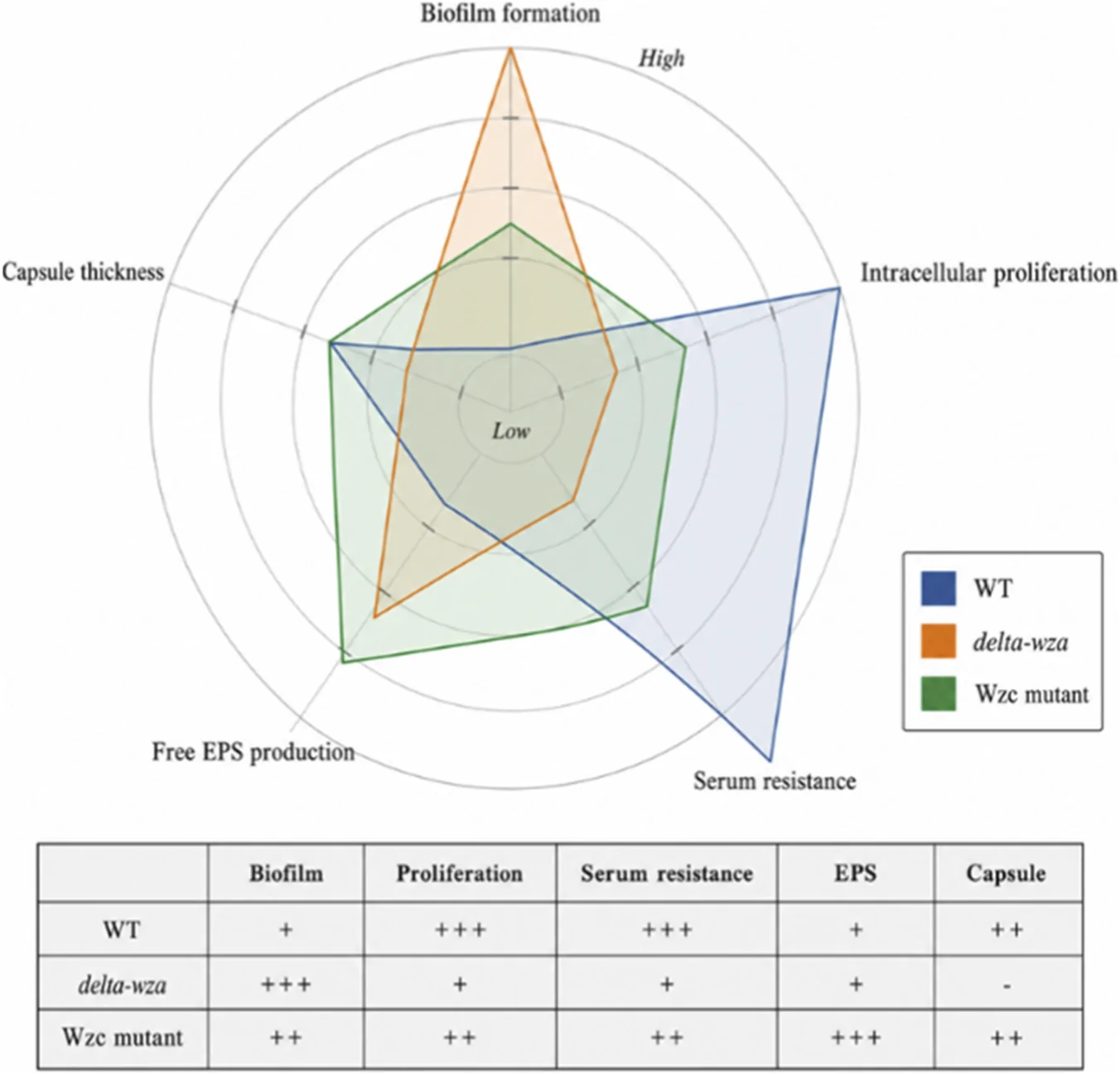
Radar chart and semi-quantitative scoring of WT, Δ*Wza*, and *Wzc* mutant strains across five virulence-associated phenotypes. The five-axis radar chart displays the phenotypic profile shifts of three strains (WT blue/Δ*Wza* orange/*Wzc* mutant green) across five dimensions: biofilm formation, 24 h intracellular proliferation, serum resistance, free EPS production, and capsule thickness. WT is prominent on the serum resistance and proliferation axes; Δ*Wza* shifts markedly toward the biofilm formation axis while the serum resistance axis contracts severely; the *Wzc* mutant dominates on the EPS production and capsule thickness axes. The lower table provides semi-quantitative scores (+++/++/+/−) for the three strains across the five phenotypes. Data sources: (Dai Y. et al., 2025); (Guerra et al., 2026).

## 3 Safety reconstitution and detoxification of the Kqp secretion chassis

### 3.1 Precision genomic erasure of virulence effectors and resistance elements

Converting Kqp from an opportunistic pathogen into an industrial secretion chassis first requires establishing efficient genome editing capability (Ring et al., 2023). The adaptation of the CRISPR-Cas9 system in *Klebsiella* has undergone iterative development from dual-plasmid to single-plasmid formats. Wang et al. (Wang et al., 2018) developed the pCasKP-pSGKP dual-plasmid system, which for the first time coupled Cas9/sgRNA-targeted cleavage with the λ-Red recombinase system, achieving scarless gene deletion in both clinical and industrial strains; the pBECKP base editor derived by the same team utilizes cytidine deaminase to achieve C→T single-base substitutions without introducing double-strand DNA breaks (Wang et al., 2018). Elsayed et al. (Elsayed et al., 2025) developed the modular single-plasmid RECKLEEN platform and reported up to 100% positive counter-selected clones for some targets and up to 72% simultaneous multi-target deletion in the tested *K. pneumoniae* strains. Collias et al. (Collias et al., 2023) used attenuated guide RNAs to reduce lethal targeting while retaining editing activity across the bacterial hosts tested; this strategy is comparative evidence rather than a Kqp-validated platform (Table 1; Table 2). Figure 7 summarizes the source hosts, molecular operations, and reported endpoints of these CRISPR tools in a qualitative evidence matrix. Baba et al. (Baba et al., 2026) encountered strain-specific obstacles when attempting to construct CRISPR plasmids for five MDR clinical isolates, suggesting that the universality of editing tools across different *Klebsiella* strain backgrounds requires strain-specific validation, and direct transfer to Kqp remains untested (Li et al., 2025).

**TABLE 2:** Systematic comparison of CRISPR genome editing tools for *K. pneumoniae* (including plasmid systems, Cas9 variants, editing types, efficiency, PAM requirements, Kqp suitability, and 11 attributes in total). References: (Wang et al., 2018; Elsayed et al., 2025; Yao et al., 2022; Baba et al., 2026).

Attribute	pCasKP-pSGKP	pBECKP	RECKLEEN	Compact CRISPRi	References
Experimental host	Industrial and clinical *K. pneumoniae* strains	Clinical *K. pneumoniae* strains	*K. pneumoniae* ATCC 700721, BAA-1705, 700603 and related strains	ST23 *K. pneumoniae* isolate; pathogenic *E. coli* extension	(Wang et al., 2018; Elsayed et al., 2025; Yao et al., 2022)
Molecular effector	SpCas9 + -Red	APOBEC1–nCas9(D10A)	Cas9 or SpG Cas9 + -Red + AcrIIA4	dCas9 + sgRNA	(Wang et al., 2018; Elsayed et al., 2025; Yao et al., 2022)
Operation	Scarless deletion and iterative editing	C→T base editing in chromosomal/plasmid genes	Deletion, point mutation, DNA integration, multi-target deletion	Transcriptional repression of natural plasmid-borne genes	(Wang et al., 2018; Elsayed et al., 2025; Yao et al., 2022)
DNA cleavage	Double-strand break	Single-strand nick; no DSB	Double-strand break with counterselection	No DNA cleavage	(Wang et al., 2018; Elsayed et al., 2025; Yao et al., 2022)
Source-reported outcome	Several single-locus deletions reached 10/10 or 20/20 positive clones; locus dependence was also reported	*fosA* stop-codon editing 8/8; blaCTX-M-65 example 2/8	Up to 100% of counter-selected clones; simultaneous multi-target deletion up to 72%	blaNDM-1 repression reduced meropenem MIC >1,000-fold; dual targeting altered two -lactam MICs	(Wang et al., 2018; Elsayed et al., 2025; Yao et al., 2022)
Selection/counterselection	Cas9 cleavage + -Red; sacB-assisted plasmid curing	Base editing; sacB-assisted plasmid curing	Cas9/SpG counterselection modulated by AcrIIA4	Inducible dCas9 repression	(Wang et al., 2018; Elsayed et al., 2025; Yao et al., 2022)
Targeting constraint	SpCas9 PAM	SpCas9 PAM and base-editing window	SpCas9 or near-PAMless SpG variant	sgRNA target compatible with dCas9	(Wang et al., 2018; Elsayed et al., 2025; Yao et al., 2022)
Multiplex capability	Iterative editing	Iterative base editing	Simultaneous two- or three-target deletion reported	Simultaneous repression of blaNDM-1 and blaSHV-12	(Wang et al., 2018; Elsayed et al., 2025; Yao et al., 2022)
Measured endpoint	Positive edited clones	Sequence-confirmed base conversion	Counterselection/editing frequency	Gene repression and antibiotic MIC	(Wang et al., 2018; Elsayed et al., 2025; Yao et al., 2022)
Technical limitation reported	Locus- and strain-dependent outcomes	Editing-window/context dependence	Endpoint depends on donor DNA, target and counterselection	Repression rather than permanent genome editing	(Wang et al., 2018; Elsayed et al., 2025; Yao et al., 2022)
Evidence boundary	Direct *K. pneumoniae* evidence; no Kqp experiment in source	Direct *K. pneumoniae* evidence; no Kqp experiment in source	Direct *K. pneumoniae* evidence; no Kqp experiment in source	Direct *K. pneumoniae* evidence; no Kqp experiment in source	(Wang et al., 2018; Elsayed et al., 2025; Yao et al., 2022; Baba et al., 2026)

**FIGURE 7:**
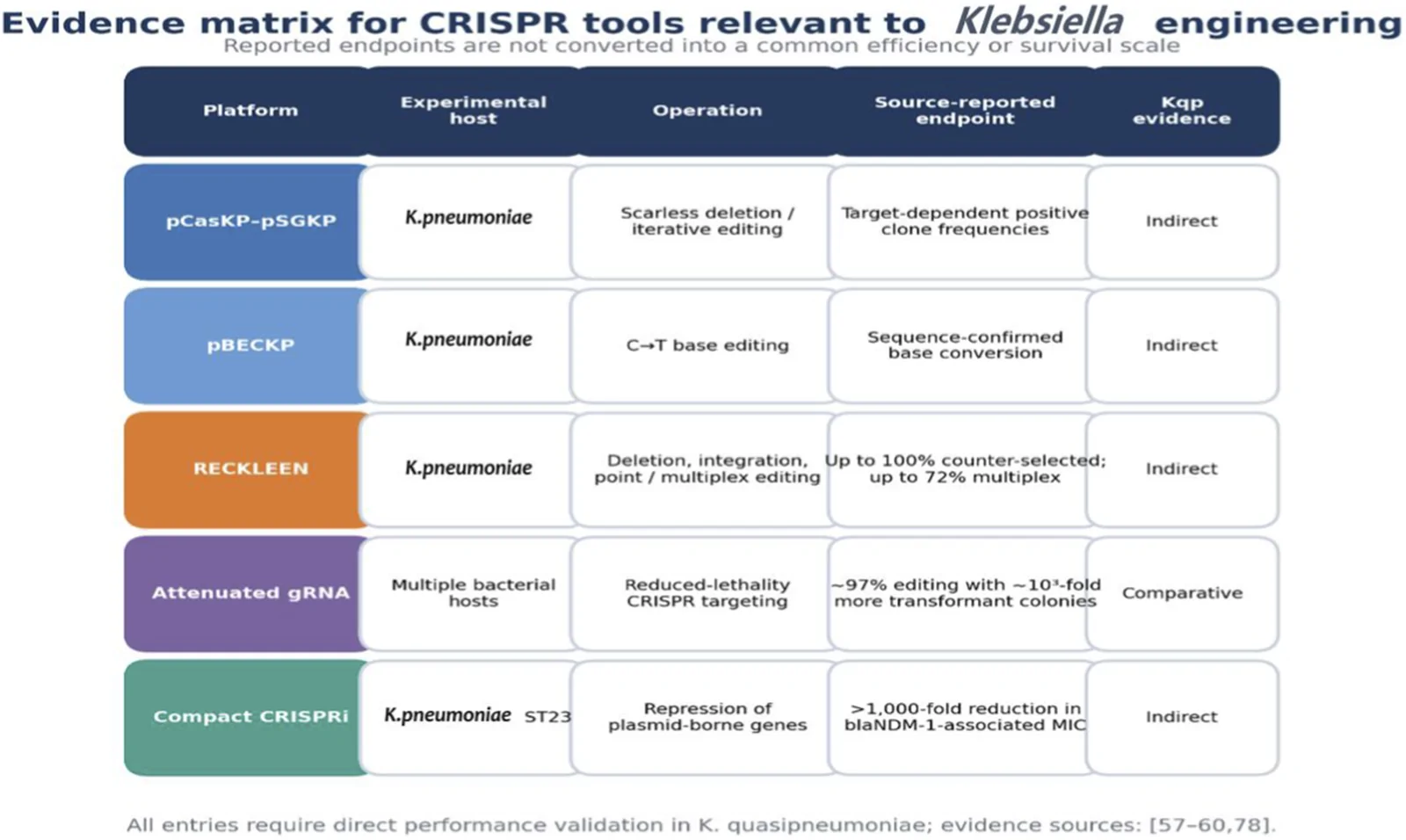
Qualitative evidence matrix of CRISPR tools. Rows summarize pCasKP-pSGKP, pBECKP, RECKLEEN, attenuated gRNA targeting, and compact CRISPRi. Columns identify the experimental host, molecular operation, source-reported endpoint, and evidence scope for Kqp. The endpoints retain their original study context and are not placed on a common efficiency or survival scale. Data sources: (Wang et al., 2018; Elsayed et al., 2025; Collias et al., 2023; Yao et al., 2022; Baba et al., 2026).

Potential detoxification targets can be discussed in three priority tiers. The first tier comprises in-frame deletion of T6SS effector-immunity gene pairs (the tae1–tae4 and tle1–tle5 families), with the proposed aim of reducing bactericidal activity; the cited studies establish contact-dependent competition but do not validate this engineering outcome in Kqp (Bray et al., 2025; Li W. et al., 2024; Storey et al., 2020; Mohamed et al., 2023; Liu et al., 2023; Zhang et al., 2022; Liu et al., 2017; Haddadi et al., 2025; Zhu et al., 2025). The second tier involves targeted elimination of plasmid-encoded carbapenemase and ESBL genes (*blaKPC-2*, *blaNDM-1*, *blaOXA-48-like*, *blaCTX-M*, *blaSHV*), for which editing or transcriptional repression has been reported in *K. pneumoniae* systems (Wang et al., 2018; Elsayed et al., 2025; Yao et al., 2022; Baba et al., 2026). The third tier is the deletion of siderophore biosynthesis gene clusters (aerobactin *iucABCD*–*iutA* and yersiniabactin *irp1*–*irp2*–*ybtA*–*ybtS*), whose deletion effects remain host- and medium-dependent (Bray et al., 2025; Paczosa and Mecsas, 2016; Russo and Marr, 2019). It must be particularly emphasized that the AcrAB-TolC and OqxAB efflux pumps, while functioning as resistance factors in clinical settings, are primarily documented as antimicrobial-resistance or stress-associated systems; direct transport of 1,3-PDO or 2,3-BDO has not been demonstrated (Bhogal et al., 2025; Gravey et al., 2024; Zhang et al., 2023). These two categories of efflux pumps are therefore discussed through a proposed, unvalidated regulatory-decoupling concept: preserve the coding sequences while placing *ramA* under the control of a product-responsive or growth-phase-dependent promoter (Gravey et al., 2024; Nasralddin et al., 2025). As an orthogonal safeguard against residual pathogenicity, phage-based interventions remain promising, although regulatory standardization and implementation barriers remain substantial (Gallina et al., 2026).

### 3.2 Substrate specificity redirection of secretion channels

Upon completing the removal of virulence effector proteins and resistance elements, the liberated secretion channel capacity must be reallocated to target industrial products. For T2SS-mediated protein secretion, signal-peptide optimization addresses inner-membrane targeting but does not alone determine T2SS outer-membrane substrate recognition (Karyolaimos and de Gier, 2021; Eom et al., 2024; Ghasemi et al., 2022; Zhang and Duan, 2023). Eom et al. (Eom et al., 2024) demonstrated through comparative analysis that a variant signal peptide of *B. subtilis* chitosanase lacking six amino acids in its native N-region (mCsn2-SP) achieved a 2.37-fold increase in secretion efficiency over the full-length signal peptide and successfully mediated the periplasmic translocation of human Cu,Zn-SOD. PulA studies in *K. oxytoca* and *K. pneumoniae* provide a comparative T2SS benchmark rather than direct Kqp validation (Li Y. et al., 2023; Cianciotto and White, 2017; Nivaskumar and Francetic, 2014; Pugsley et al., 1986). The molecular chaperone co-overexpression strategies reviewed by Karyolaimos et al. (Karyolaimos and de Gier, 2021) — encompassing Skp, SurA, FkpA, and the DsbA-DsbC oxidoreductase isomerase system—may inform future Kqp work but have not been validated in Kqp, although the activation thresholds of the Cpx and σ^E^ envelope stress pathways of Kqp have not yet been experimentally calibrated (Raivio et al., 2013; Grabowicz and Silhavy, 2017). Furthermore, endogenous T2SS substrates occupy a substantial fraction of translocon capacity and pseudopilus binding sites; graded knockdown is a proposed strategy that could release flux capacity while avoiding the triggering of periplasmic protease degradation cascades (Zhang and Duan, 2023).

### 3.3 Introduction and multi-layered architecture of biocontainment systems

The ultimate safety closure of an industrial Kqp chassis depends on a multi-layered biocontainment system (Table 3). The first layer consists of metabolic auxotrophies: auxotrophic and logic-gated systems report host- and assay-specific escape endpoints that should not be treated as a universal Kqp value (Chan et al., 2016; Eckerstorfer et al., 2025; Asin-Garcia et al., 2022); the phosphite synthetic auxotrophy developed by Asin-Garcia et al. (Asin-Garcia et al., 2022) exploits a reduced phosphorus compound not found in nature to achieve fully orthogonal metabolic dependence. The second layer comprises programmed kill switches: Maneira et al. (Maneira et al., 2025) developed a dual-input transcriptional kill-switch system (aTc-dependent + temperature-sensitive) in yeast that maintained an escape frequency below 10^–9^ CFU^−1^ for over 6 days under restrictive conditions while remaining stable for more than 100 generations under permissive conditions. Cas9-assisted containment was reported in *Bacteroides* (Hayashi et al., 2024), whereas a separate multi-target CRISPR kill switch was evaluated in *E. coli* Nissle (Rottinghaus et al., 2022). The third layer uses an estradiol-regulated ERdd protein-stability switch in *S. cerevisiae*: estradiol stabilizes essential-protein fusions, whereas its withdrawal promotes their degradation (Hoffmann and Cai, 2024). Because these systems were measured in different hosts and assays, their escape probabilities cannot be multiplied into a validated Kqp containment value. It should be noted that Asin-Garcia et al. (Asin-Garcia et al., 2023) systematically mapped the knowledge and action gaps in this field through a value-sensitive design approach, revealing that the industrial deployment of biocontainment systems substantially lags behind academic proof-of-concept demonstrations (Asin-Garcia et al., 2020).

**TABLE 3:** Source-specific biocontainment strategies and reported experimental endpoints. Values retain the original host and assay and are not multiplied into a Kqp regulatory threshold. References: (Chan et al., 2016; Eckerstorfer et al., 2025; Asin-Garcia et al., 2022; Maneira et al., 2025; Hayashi et al., 2024; Rottinghaus et al., 2022; Hoffmann and Cai, 2024); (Stirling et al., 2017).

Strategy	Category	Mechanism	Source-reported endpoint	Inducer required	Stability (permissive)	Evidence scope	Limitations	References
*dapA* (source-host benchmark)	Metabolic auxotrophy	DAP-dependent (cell wall precursor)	Not reported in cited sources	No	>100 generations	High (mature technology)	Single marker escape rate insufficient	(Eckerstorfer et al., 2025); (Asin-Garcia et al., 2022)
*dapA* + *thyA* (literature proposal)	Double auxotrophy	Two orthogonal metabolic blocks	Not reported in cited sources	No	Stable	High	Slightly increased medium cost	(Eckerstorfer et al., 2025); (Asin-Garcia et al., 2022)
Phosphite auxotrophy (*P. putida*)	Synthetic auxotrophy	Phosphite substitutes phosphate (absent in nature)	2.05 × 10^−10^ ± 2.76 × 10^−10^ (21 days; soil-extract agar)	Phosphite supplementation required	Stable	Moderate–high	Phi cost; metabolic burden	(Asin-Garcia et al., 2022)
Deadman (*E. coli*)	Toxin-antitoxin	aTc-induced antitoxin; ccdB lethal upon withdrawal	∼10^−7^–10^–8^	Yes (aTc)	>100 generations (with aTc)	Moderate	Single-layer escape rate insufficient; continuous aTc required	(Chan et al., 2016)
Passcode (*E. coli*)	Logic-gate integration	aTc + IPTG + temperature ≥3 signals required for survival	<10^–10^	Yes (multiple signals)	>100 generations (fully permissive)	Moderate	Multiple inducer costs uneconomical	(Chan et al., 2016)
Two-input containment (*S. cerevisiae*)	Dual-input kill switch	Tet-On + temperature-sensitive CcdB	<10^–9^ CFU^−1^ (6 days)	Yes (aTc)	>100 generations (37 °C + aTc)	High	Validated in eukaryotes; bacteria pending	(Maneira et al., 2025)
Cas9-assisted/CRISPR containment (*Bacteroides*; *E. coli* Nissle)	Programmable CRISPR	Cas9 induction → cleavage of *dnaA*/*ftsZ* (multiple essential genes)	Source-specific *in vitro*/*in vivo* killing or containment endpoints	Yes (signal absence)	Stable (pre-induction)	High	Induction requires fine design	(Hayashi et al., 2024); (Rottinghaus et al., 2022)
Protein-stability switch (*S. cerevisiae*)	ERdd protein-stability switch	Estradiol-dependent stability of an essential protein	SPC110-ERdd: <5 × 10^−7^; optimized SPC110Δ845-ERdd: 8.4 × 10^−9^; dual ERdd switch: approximately <2 × 10^−10^	Yes (estradiol)	Assessed over 100 generations under permissive conditions	Yeast source-host evidence	Estradiol-dependent; endpoint varies by construct	(Hoffmann and Cai, 2024)

## 4 Secretion mechanism reprogramming and synthetic biology enabling technologies

### 4.1 Signal peptide engineering: Library construction and high-throughput screening

The extracellular secretion efficiency of recombinant proteins in a prospective Kqp system may be influenced by the product of three serial bottlenecks: inner membrane translocation rate (Sec/Tat pathways), periplasmic folding efficiency (molecular chaperone network), and T2SS outer membrane extrusion throughput (pseudopilus dynamics) (Karyolaimos and de Gier, 2021; Eom et al., 2024). The signal peptide, as the entry determinant for proteins entering the secretory pathway, exhibits differential sequence preferences in its tripartite structure across different species and SecYEG translocon subtypes (Eom et al., 2024; Ghasemi et al., 2022). Eom et al. (Eom et al., 2024) showed that a variant signal peptide with a six-amino-acid N-region deletion achieved a 2.37-fold increase in secretion efficiency relative to the full-length signal peptide under identical conditions. As a forward-looking proposal rather than a validated Kqp workflow, a screening strategy could involve: genome-wide prediction of candidate N-terminal signal-peptide sequences using SignalP 6.0 and TatP; translational fusion with a reporter protein; and high-throughput activity screening via microfluidic fluorescence droplet sorting or FACS (Karyolaimos and de Gier, 2021; Eom et al., 2024; Teufel et al., 2022). Neural-network-based design remains a prospective direction; direct Kqp validation is not reported in the cited signal-peptide literature (Wu et al., 2020).

The practice of signal peptide engineering across diverse industrial chassis provides a comparative methodological framework for related research in Kqp. In *E. coli*, Karyolaimos et al. (Karyolaimos and de Gier, 2021) systematically reviewed the combined effects of signal peptide optimization, molecular chaperone co-overexpression, and translocon engineering on periplasmic recombinant protein yields, noting that the expression level of the SecYEG translocon is not the rate-limiting step in most cases; rather, the post-translational processing efficiency of the signal peptide (cleavage rate by signal peptidase I) and the folding kinetics of the substrate protein in the periplasm constitute the principal flux control points. Eom et al. (Eom et al., 2024), in a cross-species comparison between *B. subtilis* and *E. coli*, achieved a 2.37-fold increase in secretion efficiency through truncation engineering of the N-region of the chitosanase native signal peptide (deletion of six amino acid residues) and successfully employed this engineered signal peptide to mediate the periplasmic translocation of human Cu,Zn-SOD. This result indicates a functional coupling between N-region length and hydrophobicity on the one hand and the gating kinetics of the SecYEG channel on the other, but it does not establish transferability to Kqp. At the computational tool level, deep learning models such as SignalP 6.0 (Teufel et al., 2022) and DeepSP can now predict Sec-dependent and Tat-dependent signal peptides genome-wide with high accuracy, while attention-based neural networks have been used for *de novo* signal-peptide generation (Wu et al., 2020): their use for Kqp signal-peptide design remains prospective rather than experimentally established. The above tools and strategies have not yet been systematically validated in *Klebsiella*; the SecYEG subtype composition of Kqp, the substrate specificity of its signal peptidase I, and the compatibility of the T2SS pseudopilus with heterologous signal peptides all lack experimental data, constituting the principal knowledge gap for migrating signal peptide engineering strategies from other chassis to Kqp. Dynamic-control strategies reported in other hosts include optogenetic feedback on the unfolded protein response, quorum-sensing-controlled CRISPRi, small-molecule biosensors, and metabolite-responsive flux regulation, all of which can help decouple production from growth (Benisch et al., 2023; Zheng et al., 2025; Chaisupa and Wright, 2024; Zhou et al., 2026).

### 4.2 Industrial utilization of the type II secretion system

Structural studies of the PulL-PulM periplasmic interface provide a mechanistic reference for T2SS assembly, but the cited work does not show that altering this interface increases Kqp secretion throughput (Li Y. et al., 2023; Cianciotto and White, 2017; Nivaskumar and Francetic, 2014). Co-overexpression of periplasmic molecular chaperones (Skp, SurA, FkpA, and the DsbA-DsbC system) has been established as a core strategy for periplasmic recombinant protein production in *E. coli* in the review by Karyolaimos et al. (Karyolaimos and de Gier, 2021), and the Kqp genome encodes orthologs of all of the above chaperones. Guilvout et al. (Guilvout et al., 2024) combined structural modeling and functional analyses to support a trimeric PulF channel essential for endopilus assembly and protein secretion in the *Klebsiella* T2SS.

Quantitative characterization of T2SS throughput benefits from mature methodological accumulation across multiple systems. Li et al. (Li Y. et al., 2023) characterized the PulL-PulM periplasmic interaction using bacterial two-hybrid and functional assays, establishing its importance for secretion. The cited study does not demonstrate that interface engineering increases secretion throughput in Kqp. In *B. subtilis*, Picart et al. (Ferrando et al., 2024) employed the CRISPR-Cas9 system to systematically modulate the entire Sec secretion pathway—including overexpression of signal peptidase SipS, enhanced expression of the SecA ATPase, and deletion of cell wall-binding proteins—and the resulting super-secreting strains achieved gram-scale industrial enzyme production, illustrating a strategy in the reported *B. subtilis* host rather than direct transferability to Kqp.

With respect to chaperone-assisted folding, Karyolaimos et al. (Karyolaimos and de Gier, 2021) reviewed chaperone-assisted folding strategies in *E. coli*; their quantitative performance and transferability to Kqp remain untested. The extent to which these engineering lessons from *E. coli* and *B. subtilis* are applicable to the *Klebsiella* T2SS platform remains to be experimentally evaluated; fundamental data on the activation thresholds of the Cpx and σ^E^ envelope stress pathways of Kqp, the kinetic parameters of the PulE ATPase, and the fraction of translocon capacity occupied by endogenous T2SS substrates are currently absent from the literature (Karyolaimos and de Gier, 2021; Raivio et al., 2013; Grabowicz and Silhavy, 2017). The cited Cpx and σ^E^ studies provide envelope-stress context from established bacterial models rather than measured Kqp thresholds (Raivio et al., 2013; Grabowicz and Silhavy, 2017).

### 4.3 Efflux pump engineering and enhancement of host stress tolerance

The tunability of the RamA-RamR regulatory axis — 13.1% of clinical ESBL-producing *K. pneumoniae* spontaneously carry RamR-inactivating mutations (Gravey et al., 2024) — shows regulatory variability but does not establish product-responsive pump control. Placing *ramA* under the control of a product concentration-responsive promoter is a proposed strategy that would require validation of efflux pump upregulation upon product accumulation (Gravey et al., 2024; Nasralddin et al., 2025). Practice in *Pseudomonas* provides a cross-species comparison: Cárdenas Espinosa et al. (Cárdenas Espinosa et al., 2023) demonstrated that the genome-reduced *Pseudomonas* taiwanensis GRC3, which retains the TtgGHI RND pump, exhibited an EC50 for 1-alkanols twice that of *P. putida* DOT-T1E. The cryo-EM structure of KpAcrB determined by Zhang et al. (Zhang et al., 2023) defines an antibiotic-bound structural reference but does not establish diol transport or substrate-selective redesign: the hydrophobic pocket residues (F136, F178, F614, F627, V671) in the drug-binding pocket form a hydrophobic trap that recognizes structurally diverse substrates, and molecular docking-guided site-directed mutagenesis could in principle achieve substrate spectrum remodeling of the efflux pump (Padilla et al., 2010).

Engineering of efflux pump substrate specificity has progressed from proof-of-concept to application in multiple systems. Gravey et al. (Gravey et al., 2024) provided population-genetic evidence for the natural tunability of the RamA-RamR regulatory axis through combined genomic-transcriptomic analysis of 443 clinical strains: 13.1% of ESBL-producing *K. pneumoniae* spontaneously carry functionally inactivating mutations in RamR, and the heterogeneous distribution of these mutations—encompassing frameshifts, nonsense mutations, and ligand-binding domain missense substitutions—indicates that the DNA-binding and ligand-sensing functions of RamR are evolutionarily separable, showing regulatory diversity without demonstrating product-responsive activation. In *Pseudomonas*, Cárdenas Espinosa et al. (Cárdenas Espinosa et al., 2023) systematically evaluated the tolerance of eight genome-reduced *P. putida* and P. taiwanensis strains to ten organic solvents, finding that P. taiwanensis GRC3, which retains the TtgGHI RND pump, exhibited an EC50 for 1-alkanols twice that of *P. putida* DOT-T1E, supporting a host-specific association between the retained RND system and solvent tolerance of genome-reduced strains. At the structural level, the cryo-EM structure of KpAcrB (PDB 8FFS) determined by Zhang et al. (Zhang et al., 2023) revealed a hydrophobic trap composed of five hydrophobic pocket residues—F136, F178, F614, F627, and V671 — in the distal binding pocket; Padilla et al. (Padilla et al., 2010) demonstrated contributions of AcrAB to antimicrobial resistance and virulence in *K. pneumoniae*. Building on this structural information, Wilhelm and Pos (Wilhelm and Pos, 2024) reviewed structural determinants of substrate specificity and inhibition in AcrB and AdeB. At the regulatory level, Teng et al. (Teng et al., 2024) investigated and engineered a 1,2-propanediol-responsive PocR biosensor, providing a precedent for tuning ligand responsiveness. Because the PocR study concerns 1,2-propanediol sensing, its use for 1,3-PDO- or 2,3-BDO-responsive ramA control remains a proposal rather than a demonstrated transfer (Teng et al., 2024). The most prominent knowledge gap in this field at present is that the transport kinetic parameters (Km and Vmax) of AcrAB-TolC and OqxAB for 1,3-PDO and 2,3-BDO have not been directly measured (Bhogal et al., 2025; Gravey et al., 2024; Zhang et al., 2023), limiting the quantitative assessment of the contribution of efflux pumps to product tolerance under industrial fermentation conditions.

It merits emphasis that the prerequisite for implementing all of the above engineering strategies is efficient Kqp genome editing capability. Figure 7 summarizes CRISPR tools reported in *K. pneumoniae* or other bacterial hosts as a qualitative evidence matrix (Wang et al., 2018; Elsayed et al., 2025; Collias et al., 2023; Yao et al., 2022). The pCasKP-pSGKP and pBECKP systems provide gene deletion and base-editing functions, respectively (Wang et al., 2018). The RECKLEEN single-plasmid platform supports several editing operations with target-dependent outcomes (Elsayed et al., 2025). Collias et al. (Collias et al., 2023) attenuated gRNA targeting to reduce lethal cleavage while retaining editing activity; Yao et al. (Yao et al., 2022) developed a compact CRISPRi system for repression of plasmid-borne genes. The different source hosts and measured endpoints preclude a common efficiency-survival ranking. Baba et al. (Baba et al., 2026) encountered strain-specific obstacles when attempting to construct CRISPR plasmids for five MDR clinical isolates, suggesting that the universality of editing tools across different *Klebsiella* strain backgrounds requires strain-specific validation, and direct Kqp performance remains untested (Li et al., 2025).

### 4.4 Chassis engineering of outer membrane vesicles

After completing the removal of pathogenic cargoes, OMVs can be positively engineered into vaccine delivery vehicles, drug nanocarriers, or nanobioreactors (Weyant et al., 2023; Szöllősi et al., 2023; Wilhelm and Pos, 2024; Varol et al., 2025; Garling et al., 2025; Ruiz-Moreno et al., 2025). Weakening of *tolA*, *tolR*, or *pal*, or deletion of *lpp*, can increase OMV production by 10- to 50-fold (Dell'Annunziata et al., 2021; Varol et al., 2025; Garling et al., 2025; Ruiz-Moreno et al., 2025). Ruiz-Moreno et al. (Ruiz-Moreno et al., 2025) achieved enhanced OMV production in *E. coli* through genome-scale metabolic model (GEM)-guided lipidome optimization, a framework that may inform future Kqp studies but has not been validated in Kqp. Target protein loading follows three non-mutually exclusive routes: stochastic encapsulation following periplasmic enrichment; surface display via genetic fusion with outer membrane proteins (OmpA, OmpW, ClyA); and passive loading of purified OMVs by *in vitro* electroporation (Jiang et al., 2022; Weyant et al., 2023; Szöllősi et al., 2023; Li Q. et al., 2023). Jiang et al. (Jiang et al., 2022) displayed the SARS-CoV-2 RBD on the surface of *Salmonella* OMVs via SpyTag-SpyCatcher coupling, and Besançon et al. (Besançon et al., 2026) built upon this by introducing a STING-adjuvanted core to achieve heterologous Kp capsule cross-protection. The core bottleneck in current OMV engineering lies in batch-to-batch heterogeneity—size distribution and cargo loading vary across preparations and require source-specific quality assessment—and the genetic factors governing these parameters do not yet support quality-by-design-based homogeneous production (Garling et al., 2025). Increased vesicle yield does not imply reduced endotoxin: OMV quantity, cargo loading, LPS content, and inflammatory activity are separate quality attributes (Hussein et al., 2023; Dell'Annunziata et al., 2021; Varol et al., 2025; Garling et al., 2025; Tang et al., 2023; Leitner et al., 2013; Kim et al., 2009; Ruiz-Moreno et al., 2025).

OMV engineering has accumulated systematic experience spanning from yield optimization to cargo loading across multiple systems. In terms of OMV yield enhancement, multiple independent studies consistently demonstrate that weakening of *tolA*, *tolR*, or *pal* can increase OMV production by 10- to 50-fold, with the underlying mechanism being that the Tol-Pal system maintains the physical connection between the outer membrane and the peptidoglycan layer, and weakening of this system lowers the energetic barrier for outer membrane budding (Dell'Annunziata et al., 2021; Varol et al., 2025; Garling et al., 2025); deletion of *lpp* similarly enhances OMV production by weakening the covalent anchoring of the outer membrane mediated by Braun’s lipoprotein (Ruiz-Moreno et al., 2025). Ruiz-Moreno et al. (Ruiz-Moreno et al., 2025) achieved enhanced OMV production in *E. coli* through GEM-guided lipidome optimization, a method that establishes membrane lipid composition as a key engineering target for OMV production by systematically analyzing the quantitative relationship between membrane lipid composition (ratios of phosphatidylethanolamine, phosphatidylglycerol, and cardiolipin) and OMV yield.

With respect to cargo loading strategies, the current literature reports three non-mutually exclusive routes: periplasmic enrichment followed by stochastic encapsulation is the most straightforward strategy, suitable for highly expressed soluble periplasmic proteins (Jiang et al., 2022); surface display via genetic fusion with outer membrane proteins (OmpA, OmpW, ClyA) has been successfully employed to present the SARS-CoV-2 RBD antigen on the surface of *Salmonella* OMVs (Jiang et al., 2022); and passive loading of purified OMVs by *in vitro* electroporation offers flexibility but with lower loading efficiency (Weyant et al., 2023; Szöllősi et al., 2023). Jiang et al. (Jiang et al., 2022) achieved oriented display of SARS-CoV-2 RBD on *Salmonella* OMV surfaces via the SpyTag-SpyCatcher covalent coupling system, and Besançon et al. (Besançon et al., 2026) further introduced a STING agonist-adjuvanted core on this platform, achieving cross-protection against different Kp capsule types in a lethal pneumonia model, demonstrating the modular design advantages of the OMV platform in vaccine development.

With regard to OMV product homogeneity, Garling et al. (Garling et al., 2025) noted in their review that the principal sources of batch-to-batch heterogeneity in current OMV products are size distribution, cargo loading per vesicle, and batch-to-batch fluctuation in surface antigen density, and that the genetic determinants of these parameters have been only sporadically reported—for instance, a trend toward narrowed OMV size distribution has been observed in *tolR* mutant strains, but the underlying molecular mechanism remains unelucidated. These methodological frameworks established in *E. coli* and *Salmonella* provide a comparative foundation for future Kqp OMV studies, although the characteristic high-CPS background and species-specific OMV proteome composition of Kqp may introduce additional engineering complexity and quality control challenges not yet discussed in the literature. Hypervesiculation can also increase the total LPS-bearing particle burden; detoxification of lipid A/LPS and vesicle-yield optimization should therefore be evaluated independently.

## 5 Frontier application prospects of the Kqp secretion chassis

### 5.1 Bulk chemicals and stress-tolerant fermentation

In the *Klebsiella* strains studied, the metabolic architecture of glycerol dissimilation features an oxidative branch that supplies ATP, NADH, and biomass precursors, and a reductive branch that employs 1,3-PDO as the terminal electron acceptor for NADH reoxidation—an evolutionarily elegant redox self-balancing system (Oh et al., 2018; Ma et al., 2019; Jo et al., 2024a; Lee et al., 2018; Sun et al., 2022; Wang et al., 2021; Lin et al., 2023; Li L. et al., 2024; Tao et al., 2019). Oh et al. (Oh et al., 2018) employed a lactate-deficient *K. pneumoniae* ATCC 200721 mutant (ldhA) in repeated fed-batch fermentation to achieve a 1,3-PDO titer of 81.1 g/L and a volumetric productivity of 3.38 g/L/h, with no performance deterioration across five consecutive fermentation cycles (Lee et al., 2018; Sun et al., 2022). Ma et al. (Ma et al., 2019) isolated strain 2e, representing a genetic-engineering-independent alternative strategy: the 1,3-PDO yield reached 0.64 mol/mol glycerol, and comparative genomics identified a novel glycerol uptake facilitator protein and an expanded repertoire of stress response proteins (Wang et al., 2021; Lin et al., 2023; Li L. et al., 2024). Figure 8 presents, in bar chart format, a summary of the titer distribution landscape of 1,3-PDO and 2,3-BDO production strains reported in the literature. From the figure, it can be clearly seen that the density curve for 1,3-PDO production strategies shifts rightward layer by layer, across source-specific *K. pneumoniae* strains and cultivation modes; the values should not be read as a controlled ranking of engineering complexity. For 2,3-BDO, *K. pneumoniae* GEM167 derivatives produced 89.47 ± 0.11 g/L (R, R)-2,3-BDO and 105.3 ± 2.8 g/L total 2,3-BDO under the respective fed-batch conditions (Jo et al., 2024a; Jo et al., 2024b). Conditional overexpression of the native *budABC* in Kqp, coupled with product-responsive upregulation of AcrAB-TolC, is a forward-looking hypothesis that has not been experimentally validated in Kqp, benchmarked against 1,3-PDO titer levels (Jo et al., 2024b; Zhang X. et al., 2025) (Table 4). A critical fundamental knowledge gap is that the transport kinetic parameters (Km and Vmax) of AcrAB-TolC and OqxAB for 1,3-PDO and 2,3-BDO have not been directly measured to date, and these parameters are necessary inputs for the rational integration of efflux pump engineering into production strain development workflows (Bhogal et al., 2025; Gravey et al., 2024; Zhang et al., 2023).

**FIGURE 8:**
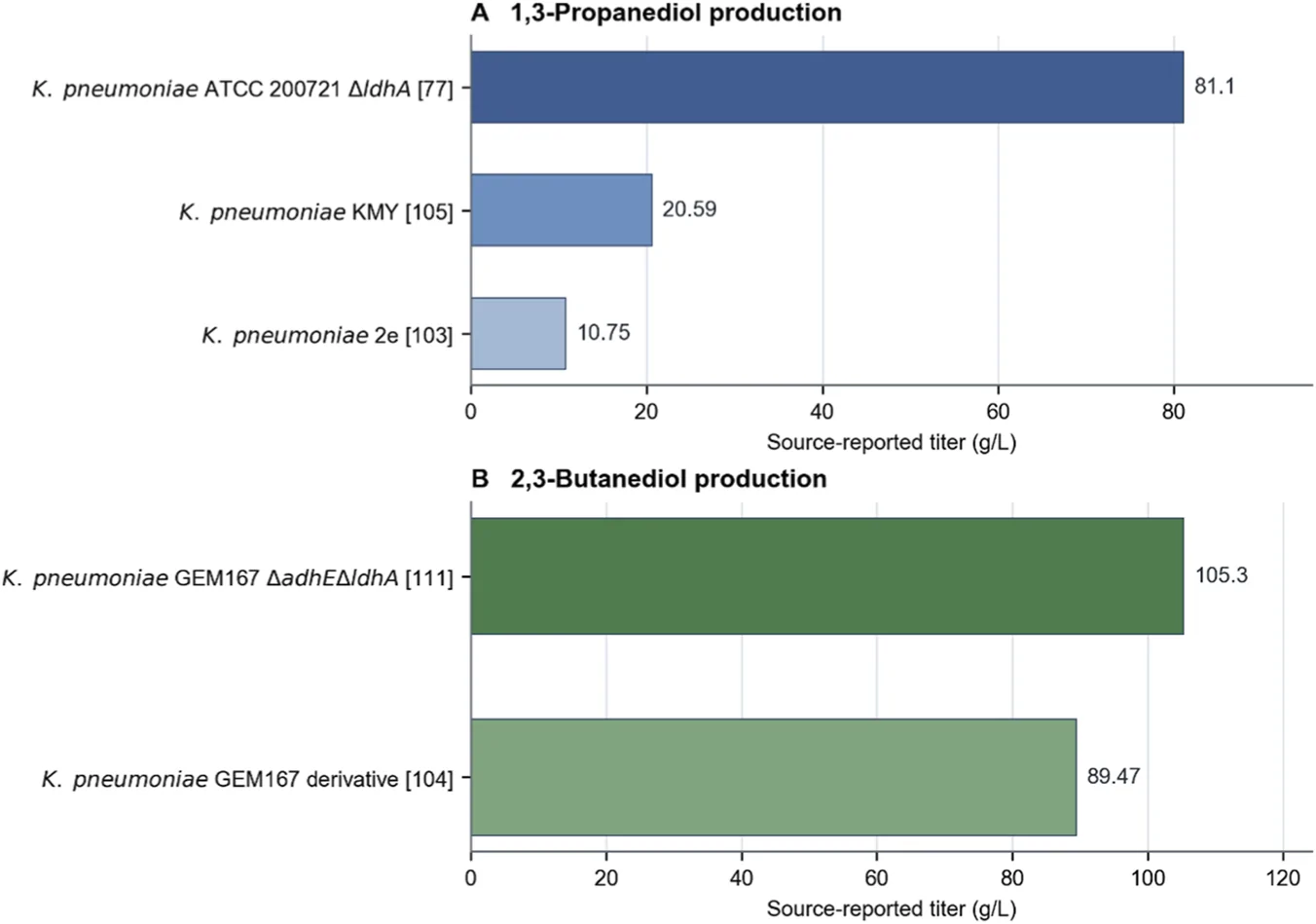
Source-reported 1,3-PDO and 2,3-BDO production in *K. pneumoniae* strains. **(A)** 1,3-PDO titers for *K. pneumoniae* ATCC 200721 *ldhA* (81.1 g/L), strain KMY (20.59 g/L), and strain 2e (10.75 g/L). **(B)** 2,3-BDO titers for *K. pneumoniae* GEM167 *adhE ldhA* (105.3 ± 2.8 g/L) and a GEM167 derivative carrying *adhE ldhA budC* with *dhaD* overexpression (89.47 ± 0.11 g/L). Values are source-specific and are not normalized across substrates or cultivation modes. Data sources: (Oh et al., 2018); (Ma et al., 2019; Jo et al., 2024a; Lee et al., 2018); (Jo et al., 2024b).

**TABLE 4:** Source-reported fermentation and bioconversion benchmarks in *Klebsiella* and other named hosts, including product, strain, genotype, substrate, cultivation mode, reported metric, yield, productivity, and evidence scope. Values retain their original units and do not establish AcrAB-TolC- or OqxAB-mediated export. References: (Oh et al., 2018); (Ma et al., 2019; Jo et al., 2024a; Lee et al., 2018); (Jo et al., 2024b; Zhang X. et al., 2025; Zhou et al., 2019; Liang et al., 2024; Zhang K. et al., 2025); (You et al., 2024); (Kumar et al., 2016). Legacy strain names, including KMY and ATCC 200721, follow the original publications; genome-based reidentification was not reported in those sources.

Product	Strain	Genotype/strategy	Substrate	Mode	Reported metric	Yield	Productivity	Evidence scope	References
1,3-PDO	*K. pneumoniae* ATCC 200721	*ldhA*; lactate-deficient	Crude glycerol	Repeated fed-batch (5 cycles)	81.1 g/L	Not reported	3.38 g/L/h	Fermentation performance	(Oh et al., 2018)
1,3-PDO	*K. pneumoniae* 2e (WGS in source)	Wild-type isolate	Crude glycerol	Batch	10.75 g/L	0.64 mol/mol glycerol	∼0.90 g/L/h	Fermentation performance	(Ma et al., 2019)
1,3-PDO	*K. pneumoniae* KMY	Metabolic-pathway deletions and regulator engineering	Glycerol + mannitol	Batch	20.59 g/L	0.76 mol/mol glycerol	0.86 g/L/h	Fermentation performance	(Lee et al., 2018)
(R,R)-2,3-BDO	*K. pneumoniae* GEM167 derivative	*adhE* *ldhA* *budC*; *dhaD* overexpression	Crude glycerol	Fed-batch; two-stage agitation	89.47 ± 0.11 g/L	0.35 g/g consumed glycerol	1.24 g/L/h	Fermentation performance	(Jo et al., 2024a)
2,3-BDO	*K. pneumoniae* GEM167 *adhE ldhA*	Competitive-pathway deletions + oxygen-transfer control	Crude glycerol	Fed-batch	105.3 ± 2.8 g/L	Not reported	2.19 ± 0.06 g/L/h	Fermentation performance	(Jo et al., 2024b)
2′-FL	*E. coli* BL21star(DE3)-derived final strain	Salvage pathway; competing-pathway deletions; expression/spatial optimization	Fucose + lactose	5-L fed-batch	59.22 g/L	95% lactose and 92% fucose molar conversion	Not reported	Non-Klebsiella benchmark	(Liang et al., 2024)
2′-FL	*E. coli* RFL38	*De novo* GDP-L-fucose pathway and flux regulation	Fructose + lactose	Fed-batch bioconversion	41.6 g/L	0.482 mol/mol fructose; 0.986 mol/mol lactose	Not reported	Non-*Klebsiella* benchmark	(You et al., 2024)
2′-FL	*E. coli* CZF derivative	*B. cereus* *FutCB*; *lacZ* *waaF*; precursor-pathway overexpression	Glycerol + lactose	10-L fed-batch	121.4 g/L	Not reported	1.90 g/L/h	Non-*Klebsiella* benchmark	(Zhang et al., 2025b)
D-tagatose	Engineered *E. coli* whole-cell cascade	Seven-enzyme cascade	Lactose (100 g/L)	Whole-cell bioconversion	16.75% conversion	Not reported	Not reported as g/L/h	Non-*Klebsiella* benchmark	(Zhang et al., 2025a)
Tannase	*K. pneumoniae* TAH10 (16S rRNA in source)	Native isolate; medium optimization	Indian gooseberry leaves	Submerged fermentation	0.033 U/mL	Not reported	Not reported	Enzyme-activity endpoint	(Kumar et al., 2016)

### 5.2 Cell factories for high-value carbohydrates

The Wzx/Wzy/Wza polysaccharide assembly–export pathway is a major native polysaccharide assembly-export system discussed for KpSC (Pan et al., 2015; Guerra et al., 2026; Xu et al., 2024; Whitfield et al., 2020). The microbial production of 2′-fucosyllactose (2′-FL) has been extensively validated in *E. coli* through multiple studies: Liang et al. (Liang et al., 2024) reported 59.22 g/L 2′-FL in a 5-L *E. coli* fed-batch process after pathway, expression, and spatial-organization optimization. Zhang et al. (Zhang K. et al., 2025) used a *Bacillus cereus* −1,2-fucosyltransferase in an engineered *E. coli* process that reached 121.4 g/L 2′-FL; related studies report additional *E. coli* or cell-free routes (Shin et al., 2022; You et al., 2024; Dutschei et al., 2022; Bai et al., 2025; Hoang et al., 2026). Figure 9 presents a schematic map of the strategy space for 2′-FL production: salvage and *de novo* pathways are linked to the source hosts and engineering strategies, including enzyme selection, precursor supply, expression tuning, and fed-batch scale-up. A prospective, unvalidated Kqp hypothesis is that its native CPS synthesis pathway has a substantial, sustained demand for nucleotide sugar precursors such as GDP-fucose and UDP-galactose; knockout of CPS entry-point genes (*wbaP* or *wcaJ*) might redirect the accumulated activated sugar precursors toward heterologous HMO production (Guerra et al., 2026; Xu et al., 2024). However, it must be prudently assessed whether blockade of CPS synthesis might, through as-yet-uncharacterized feedback mechanisms, downregulate the upstream biosynthetic flux of nucleotide sugar precursors—such metabolic compensation effects have been observed in other polysaccharide-producing strains (Dutschei et al., 2022). Multi-enzyme cascade synthesis of rare sugars (D-tagatose, D-allulose) has been validated both *in vitro* and *in vivo* (Zhang X. et al., 2025; Liu et al., 2024). The lower panel of Figure 9 distinguishes D-tagatose, D-allulose (also known as D-psicose), and D-allose. D-allulose and D-psicose are synonyms, whereas D-allose is a distinct sugar; conversion rates from different systems are not assigned a common qualitative ranking. *De novo* 2′-FL pathway engineering in *Escherichia coli* provides complementary design principles for GDP-L-fucose supply and dynamic flux balancing (Li et al., 2022).

**FIGURE 9:**
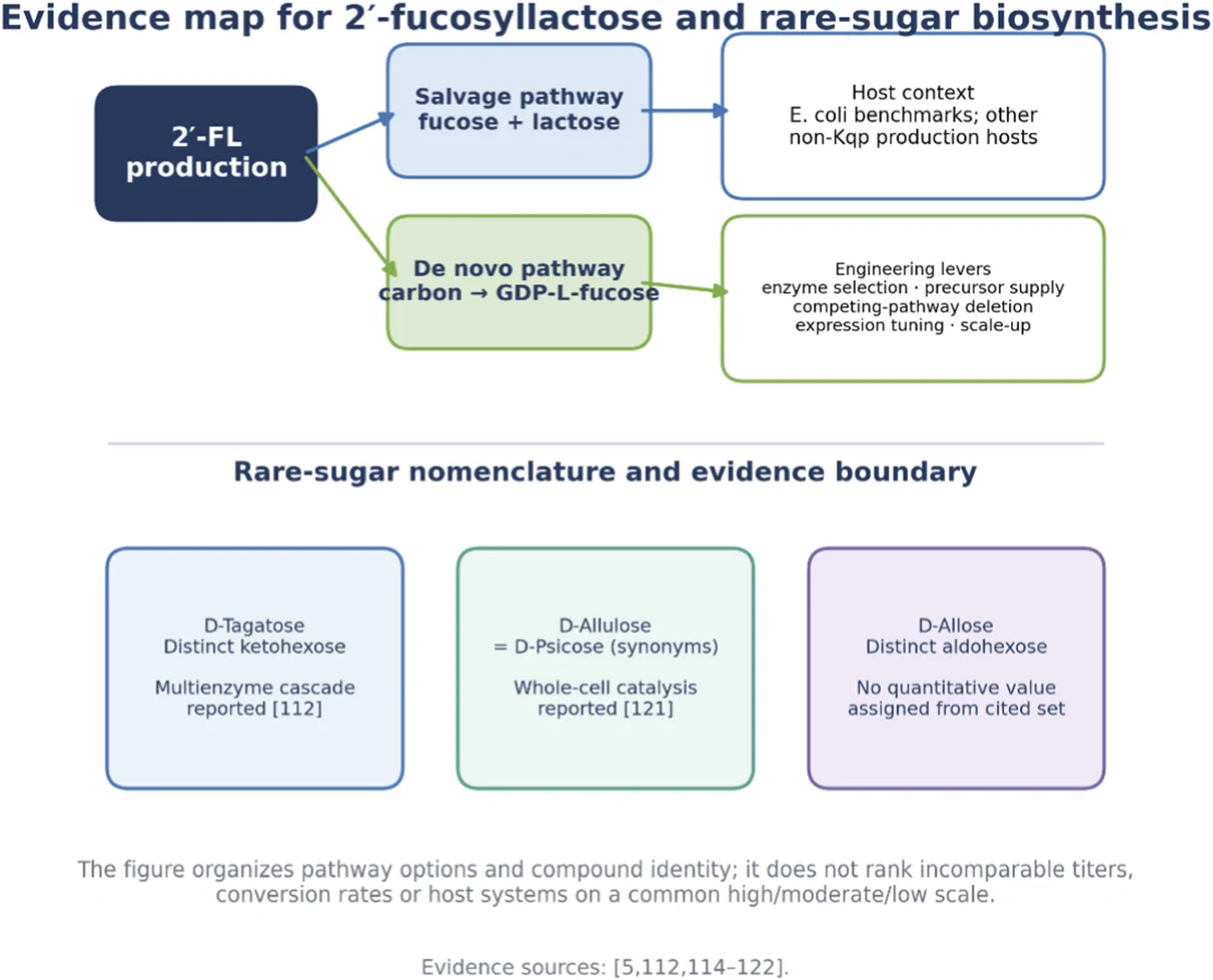
Schematic map of microbial 2′-fucosyllactose (2′-FL) production and rare-sugar identity. The upper panel links salvage and *de novo* pathways to source hosts and engineering strategies. The lower panel distinguishes D-tagatose, D-allulose (D-psicose), and D-allose. No cross-study ranking of titers or conversion rates is implied. Data sources: (Xu et al., 2021; Zhang X. et al., 2025; Liang et al., 2024; Zhang K. et al., 2025; Shin et al., 2022; You et al., 2024; Dutschei et al., 2022; Bai et al., 2025; Hoang et al., 2026; Liu et al., 2024; Li et al., 2022).

### 5.3 Recombinant proteins and industrial biologics

The well-established benchmark of efficient extracellular secretion of *K. oxytoca* PulA via T2SS (Li Y. et al., 2023; Cianciotto and White, 2017; Nivaskumar and Francetic, 2014) provides a comparative molecular framework for evaluating other proteins, but does not demonstrate their secretion by Kqp (Connor et al., 2024; Ferrando et al., 2024; Padilla et al., 2010; Kumar et al., 2016; Pang et al., 2022). Kumar et al. (Kumar et al., 2016) utilized response surface methodology to optimize submerged fermentation of *K. pneumoniae* on agricultural waste substrates for tannase production, achieving an enzyme activity of 0.033 U/mL. The tannase value is an enzyme-activity endpoint and is not directly comparable with mass titers from *Bacillus* systems; cross-host secretion-engineering results should therefore be treated as comparative precedents rather than transferable Kqp performance (Ferrando et al., 2024). Nanobodies (VHH, ∼15 kDa, single domain), by virtue of their near-instantaneous folding kinetics in the bacterial periplasm and their simple folding pathway that obviates disulfide bond isomerization, remain prospective candidates whose folding and T2SS recognition would require Kqp-specific validation (Karyolaimos and de Gier, 2021; Pang et al., 2022). The broader product landscape additionally includes neoagarooligosaccharides from engineered probiotic yeast, *Klebsiella pneumoniae*-derived ʟ-glutaminase, and biologically synthesized silver nanoparticles, providing cross-host examples in secreted enzymes and nanomaterials (Jin et al., 2021; Abdel-Hafez et al., 2025; Farsi and Metwally, 2026; Pugsley et al., 1986).

## 6 Conclusions and future perspectives

### 6.1 Four fundamental barriers from proof-of-concept to industrial fermenters

Although a preliminary literature-based framework can be outlined at the molecular level for engineering the Kqp secretion chassis, four fundamental bottlenecks constrain its translation from laboratory proof-of-concept to industrial fermenters. First, the energetic cost of secretion has not been parameterized for Kqp: each protein secretion event mediated by T2SS consumes ATP hydrolysis, superimposed on the ATP and proton motive force demands of the Sec/Tat translocons; elevated secretory flux may give rise to a secretion–growth trade-off (Karyolaimos and de Gier, 2021). Current genome-scale metabolic models (GEMs) of *Klebsiella* have not yet incorporated the energetic parameters of secretory pathways as explicit constraints. Second, the misfolding of heterologous proteins in the periplasm and their proteolytic degradation form a self-reinforcing vicious cycle: misfolded proteins activate the Cpx and σ^E^ envelope stress pathways, transcriptionally upregulating periplasmic proteases such as DegP, DegQ, and DegS, which, while degrading misfolded proteins, non-discriminately degrade correctly folded target proteins (Karyolaimos and de Gier, 2021; Raivio et al., 2013; Grabowicz and Silhavy, 2017). Sec/Tat targeting, periplasmic folding, and T2SS recognition should be considered as distinct constraints; the cited envelope-stress evidence is derived mainly from established bacterial models rather than Kqp (Karyolaimos and de Gier, 2021; Nivaskumar and Francetic, 2014; Raivio et al., 2013; Grabowicz and Silhavy, 2017). Third, batch-to-batch heterogeneity in OMV size distribution, cargo loading, and LPS-associated properties remains a product-quality concern that must be assessed with source-specific measurements (Garling et al., 2025; Ruiz-Moreno et al., 2025). Fourth, experimental validation data on real-environment escape rates of biocontainment systems are extremely scarce (Eckerstorfer et al., 2025; Asin-Garcia et al., 2022; Maneira et al., 2025; Rottinghaus et al., 2022; Hoffmann and Cai, 2024; Asin-Garcia et al., 2023; Asin-Garcia et al., 2020; Stirling et al., 2017): reported escape and stability endpoints come from different hosts and assays and therefore cannot be multiplied into a validated Kqp containment probability.

### 6.2 Quantitative measurement of secretory capacity and parameterization of the growth–secretion trade-off

Advancing the Kqp secretion chassis toward industrial application first requires answering a fundamental question: under a given genetic background and culture condition, what is the upper limit of the secretory capacity of Kqp? For literature comparison, relevant measurements can be organized conceptually spanning single cells to populations. (i) Single-cell level: single-cell reporter and microfluidic studies can quantify the protein secretion rate (molecules/cell/min) of individual Kqp cells, and describe population heterogeneity. (ii) Population level: published population-level studies can compare the protein mass distribution across four compartments—intracellular, periplasmic, OMV, and supernatant—by quantitative proteomics (TMT or LFQ labeling), while retaining each study’s original units and experimental host. (iii) Energetic level: genome-scale models may in future incorporate pathway-specific energetic costs, but no validated Kqp secretion-cost coefficients are currently available.

### 6.3 Regulatory decoupling strategies to eliminate clinical resistance risk while preserving industrial product tolerance

The functional duality of efflux pumps in clinical infection and industrial fermentation—serving as resistance factors in the former and product tolerance factors in the latter—represents the most central contradiction between the safety and industrial performance of the Kqp chassis. Resolving this contradiction cannot rely on simple gene deletion strategies; rather, it requires precise regulatory decoupling. (i) Conditional expression of AcrAB-TolC: replace the native promoters of the chromosomal *acrAB* operon and *tolC* gene with a stringent product-responsive promoter system. For 1,3-PDO production, the PocR transcription factor (reported for 1,2-propanediol sensing, not validated for 1,3-PDO) combined with the pacrA promoter (Teng et al., 2024) could be investigated as a prospective regulatory element. The original literature does not provide Kqp-specific leakage, response-threshold, or maximal-expression acceptance criteria. (ii) Substrate specificity engineering of OqxAB: by integrating information from the KpAcrB cryo-EM structure (Zhang et al., 2023), future structural models of OqxAB could be used to investigate residues in the substrate binding pockets of AcrB and OqxB that govern antibiotic recognition versus solvent recognition. Proposed replacement of putative antibiotic-recognizing residues with small hydrophobic residues (such as Ala or Gly) through site-directed mutagenesis would require assessment of the reduction in resistance levels to fluoroquinolones and macrolides using MIC plate assays and concurrent confirmation of the preservation of tolerance to 1,3-PDO and 2,3-BDO through growth inhibition experiments. (iii) Directed reconstruction of the RamA-RamR regulatory axis: while preserving the basal transcriptional repression function of RamR on RamA, altering the ligand response profile of RamR is a prospective approach that could use directed evolution of its ligand-binding domain (error-prone PCR or site-saturation mutagenesis), such that it undergoes conformational change and DNA dissociation only in response to specific industrial products rather than bile acids or antibiotics. Validate the binding affinity (Kd) and stoichiometry of engineered RamR variants with target products using surface plasmon resonance (SPR) or isothermal titration calorimetry (ITC). The strategies below are literature-informed hypotheses, not validated Kqp circuits; numerical performance targets should be defined only after host-specific measurement.

### 6.4 A regulatory-acceptable industrial safety assessment framework for Kqp

Establishing a Kqp chassis safety assessment framework aligned with applicable regulatory expectations represents a critical leap from academic concept to industrial application. This requires not only quantitative safety data but also proactive alignment with existing regulatory frameworks. (i) Genetic stability and virulence reversion risk assessment: published containment studies assess stability over source-specific passage periods; future Kqp assessment would need to monitor the genomic stability of detoxified strains using whole-genome sequencing (Illumina + Nanopore hybrid assembly), with particular attention to homologous recombination-mediated reversion of deleted virulence genes, phage-mediated horizontal gene acquisition, and IS element-mediated genome rearrangements. No universal numerical escape-rate acceptance threshold for Kqp is established by the cited literature. (ii) Environmental release safety evaluation: following OECD and EPA standard test guidelines, determine the survival curve and competition index (co-culture experiments with indigenous microbial communities) of Kqp industrial strains in simulated natural environmental matrices (soil, river water, activated sludge), and establish their decay kinetics in the absence of laboratory nutrient supplementation. Concurrently, use qPCR or ddPCR to quantify the frequency of OMV-mediated horizontal transfer of engineered DNA within environmental microbial communities, and compare with the frequencies of natural transformation and conjugation. (iii) Case-by-case regulatory assessment: given that the species identity of Kqp precludes meeting the traditional GRAS certification prerequisite of a history of safe use at the species level, the regulatory status of an engineered strain would require case-specific evaluation under the applicable framework rather than a newly asserted conditional-GRAS category. The core premise of this pathway is that safety status is determined not at the species level but on the basis of a specifically validated set of genomic modifications plus the presence and functional integrity of the requisite multi-layered biocontainment system plus product-specific toxicological and allergenicity assessment. This perspective therefore treats containment performance, environmental persistence, genomic stability, and product-specific safety as separate evidence domains (Eckerstorfer et al., 2025; Asin-Garcia et al., 2023; Asin-Garcia et al., 2020; Mec et al., 2023).

## Glossary

Kqp: *Klebsiella* quasipneumoniae
KpSC: *Klebsiella* pneumoniae
KpI: *K. pneumoniae* sensu stricto (species complex group I)
KpII: *K. quasipneumoniae* (species complex group II)
KpIII: *K. variicola* (species complex group III)
T1SS: Type I Secretion System
T2SS: Type II Secretion System
T3SS: Type III Secretion System
T4SS: Type IV Secretion System
T5SS: Type V Secretion System (Autotransporter)
T6SS: Type VI Secretion System
OMV: Outer Membrane Vesicle
OMP: Outer Membrane Protein
CPS: Capsular Polysaccharide
EPS: Extracellular Polysaccharide
LPS: Lipopolysaccharide
RND: Resistance-Nodulation-Division (efflux pump superfamily)
MFS: Major Facilitator Superfamily
MATE: Multidrug and Toxic Compound Extrusion
SMR: Small Multidrug Resistance
ABC: ATP-Binding Cassette
MDR: Multidrug Resistance
ESBL: Extended-Spectrum Beta-Lactamase
MIC: Minimum Inhibitory Concentration
CRISPR: Clustered Regularly Interspaced Short Palindromic Repeats
Cas9: CRISPR-associated Protein nine
sgRNA: Single Guide RNA
gRNA: Guide RNA
atgRNA: Attenuated Guide RNA
PCR: Polymerase Chain Reaction
1,3-PDO: 1,3-Propanediol
2,3-BDO: 2,3-Butanediol
2′-FL: 2′-Fucosyllactose
HMO: Human Milk Oligosaccharide
ATP: Adenosine Triphosphate
NADH: Reduced Nicotinamide Adenine Dinucleotide
ISPR: *In Situ* Product Recovery
DO: Dissolved Oxygen
DCW: Dry Cell Weight
CFU: Colony Forming Unit
OD600: Optical Density at 600 nm
IPTG: Isopropyl-beta-D-1-thiogalactopyranoside
TRL: Technology Readiness Level
cryo-EM: Cryo-Electron Microscopy
NMR: Nuclear Magnetic Resonance
NTA: Nanoparticle Tracking Analysis
TEM: Transmission Electron Microscopy
TMT: Tandem Mass Tag
LFQ: Label-Free Quantification
SPR: Surface Plasmon Resonance
ITC: Isothermal Titration Calorimetry
FACS: Fluorescence-Activated Cell Sorting
FBA: Flux Balance Analysis
GEM: Genome-Scale Metabolic Model
GaMD: Gaussian Accelerated Molecular Dynamics
PDB: Protein Data Bank
qPCR: Quantitative PCR
ddPCR: Droplet Digital PCR
GRAS: Generally Recognized As Safe
QPS: Qualified Presumption of Safety
FDA: U.S. Food and Drug Administration
EFSA: European Food Safety Authority
NMPA: National Medical Products Administration (China)
NIH: National Institutes of Health
OECD: Organisation for Economic Co-operation and Development
EPA: U.S. Environmental Protection Agency
SOP: Standard Operating Procedure
FDR: False Discovery Rate
ORF: Open Reading Frame
UTR: Untranslated Region
QS: Quorum Sensing
c-di-GMP: Cyclic Dimeric Guanosine Monophosphate
PAM: Protospacer Adjacent Motif
HAE: Hydrophobe/Amphiphile Efflux
